# Cultural and Developmental Influences on Overt Visual Attention to Videos

**DOI:** 10.1038/s41598-017-11570-w

**Published:** 2017-09-12

**Authors:** Omid Kardan, Laura Shneidman, Sheila Krogh-Jespersen, Suzanne Gaskins, Marc G. Berman, Amanda Woodward

**Affiliations:** 1 University of Chicago, Department of Psychology, Chicago, USA; 2 Programa Interdisciplinario sobre Política y Prácticas Educativas, Centro de Investigación y Docencia Económicas, Mexico City, Mexico; 3 DePaul University, Chicago, USA; 4 Department of Psychology, Northeastern Illinois University, Chicago, USA

## Abstract

Top-down influences on observers’ overt attention and how they interact with the features of the visual environment have been extensively investigated, but the cultural and developmental aspects of these modulations have been understudied. In this study we investigated these effects for US and Yucatec Mayan infants, children, and adults. Mayan and US participants viewed videos of two actors performing daily Mayan and US tasks in the foreground and the background while their eyes were tracked. Our region of interest analysis showed that viewers from the US looked significantly *less* at the foreground activity and spent *more* time attending to the ‘contextual’ information (static background) compared to Mayans. To investigate *how* and *what* visual features of videos were attended to in a comprehensive manner, we used multivariate methods which showed that visual features are attended to differentially by each culture. Additionally, we found that Mayan and US infants utilize the same eye-movement patterns in which fixation duration and saccade amplitude are altered in response to the visual stimuli *independently*. However, a bifurcation happens by age 6, at which US participants diverge and engage in eye-movement patterns where fixation durations and saccade amplitudes are altered *simultaneously*.

## Introduction

Tracking eye movements as a proxy for visual overt attention and studying the top-down influences on eye-movement patterns has been a subject of great interest since seminal works of Buswell^1^ and Yarbus^2^. Following Yarbus’s idea that “eye movements reflect the human thought process”, vision researchers have been investigating the top-down influence of visual tasks on overt attention^3–7^, and how the type of visual task interacts with the features of the visual stimulus^8^. However, this line of research has not investigated the influence of an observer’s cultural background on online visual attention to dynamic scenes with the same fine-grained quantitative analyses.

On the one hand researchers have made strong assumptions on the universality of basic visual attentional mechanisms (e.g. refs 9 and 10). On the other hand, it has been argued that some patterns of attention vary in fundamental ways as a function of cultural beliefs and practices (e.g. refs 11 and 12). Data from studies of overt attention during social interactions^13^ and from studies of attention to non-social scenes indicate that culture^14, 15^ or race^16^ can impact visual attention as indicated by eye-movements. However, there are other aspects of overt visual attention in relation to visual features of the environment (such as colors, edges, faces, etc.) that are assumed to be more universal^17, 18^, but might similarly vary with environmental/cultural experience. Here we ask if there are cultural differences in how low-level features (e. g., colors) and high- level features (e.g., actions) in the visual environment guide the allocation of attention as measured by eye-movement behavior. We also investigated whether these differences followed the patterns of attention allocation that have been described at more global levels of analysis. We assessed these patterns of attention across development since bifurcations due to cultural experiences are likely to develop over time.

Research in cultural psychology suggests that culture can impact how individuals attend to their environment. For example, a number of studies have demonstrated that people originating from a variety of East Asian cultures (e.g., Japanese, Koreans, and Chinese) can be more attentive to the contextual details of a scene, as well as the relationship between visual elements, while people from the US tend to focus on a central object in a visual scene (e.g. refs 15, 19–21). Some of these attentional differences are argued to derive from differences in cognitive styles (holistic vs. analytic) that are valued differently by the two cultures. As an example, Masuda and Nisbett^15^ showed that when Japanese and US participants were presented with animated vignettes of underwater scenes, participants from the US started their description of what they had seen by mentioning the salient objects in the scene while Japanese participants started by mentioning information about the field (see ref. 11 for more examples).

Others from cultural psychology have reported differences in attention between Mayan and US subjects, demonstrated in quasi-experimental and experimental work. For example, in addition to using multiple modalities to pay ‘simultaneous’ attention to multiple things (e. g., exploring a toy while visually fixated on the interviewer), Mayan toddlers and adults tend to roam their eyes to take in a lot of information at once, a visual attentional deployment that have been likened to a hummingbird’s flight pattern^22^ or an “air traffic controller’s” attentional pattern^12^. In contrast, middle-class children and adults from the United States, tend to attend to one event at a time^13^. One of the proposed reasons for this difference coming from developmental psychology has been that Mayan individuals demonstrate patterns of visual attention that are based on their regular experience as 3^rd^ party observers during their childhood^12, 22–25^. In Mayan communities, children and adult activities are integrated^13^, i. e., children most commonly learn through their participation in and observation of ongoing everyday activities in the home and the community, and they are rarely taught to do things in a direct or didactic way^24^. Thus, as compared to children growing up in WEIRD cultural communities (Western, educated, industrial, rich and democratic, see ref. 26) where engagement of adults teaching their toddlers and children by arousing their interest and refocusing their attention is frequent, Mayan children have both the opportunity and the need to learn from observation of multiple ongoing adult events^25^.

As such, attending to events that are not addressed to oneself (third-party attention) appears to be more central to learning in communities with Indigenous Mesoamerican history. However, it is important to distinguish the implications of these research findings for the current study. Third-party attention is specifically relevant for indicating *what* visual content might be more salient for Mayan participants compared to their US peers. Specifically, we expect Mayans to spend more time fixating on regions that have high content of people and activities. The second implication would be about *how* the time spent on the salient visual content is divided between fixations. Does one high priority region receive a long fixation, and then the next region receives another long fixation, or are the fixations rapidly exchanged between the two? The hummingbird flight pattern hypothesis is specifically relevant for this, since it implies larger/faster saccadic eye-movements and a higher frequency of shifting fixation points for Mayans when compared to the US viewers.

Even though these suggest that culture can exhibit top-down effects on attention, most of these studies ignore visual features and use either only gross measures of attentional allocation (i.e., whether or not the person is looking at an activity^13, 23^ or use very simple stimuli with no dynamics or social content [refs 11 and 14, see ref. 21 for more examples]. Thus, it is unknown whether these cultural differences in attentional allocation manifest in the dynamics of eye-movements in more real-world dynamic stimuli with social content, and whether they interact with the low- and high-level visual features of the scenes. In addition, while some studies suggest that culture may exert effects on visual attention as early as 4 years of age^27^, it is unknown what the trajectory of these differences looks like across development. Finally, there are disagreements as to whether observed differences in attention emerge as a result of cultural/environmental factors or apparent cultural differences are confounded by basic differences in low-level oculomotor control between groups that are not necessarily a result of differences in attention (for example see refs 28, 29 and 30). We will get back to this argument when talking about ‘phenotype’ or gene by environment effects in the discussion.

In this study we considered, across development, how participants from US and Yucatec Mayan communities deployed visual attention to dynamic social scenes. US city dwellers and Mayans from rural villages in the Yucatan watched videos that each contained 3 regions of interest (ROI’s). The first ROI, *active foreground*, consisted of an actor close to the recording camera (hence foreground) performing a daily task such as putting on socks, eating food, washing clothes, gardening, playing a game with rocks, etc. The second ROI, *active background*, consisted of another actor far from the camera (hence background) performing another daily task such as playing with toys, fixing a fence, making tortillas, etc. The third ROI, *passive background*, consisted of the remainder of the scene, which encompassed these two actors and was primarily static (typical indoors or outdoors backgrounds such as walls, floor, table, grass, trees, etc.). With this design, we were able to investigate the patterns of attentional deployment in the presence of two *competing dynamic* events (active foreground and active background) and the *static background* encompassing them. This stimulus setup approximates real-life situations better compared to static or non-social object/background stimuli and thus our results can build and extend beyond previous work. It also provides a comparison of foreground and background that does not also vary in terms of being action-based vs. static.

**Figure 1:**
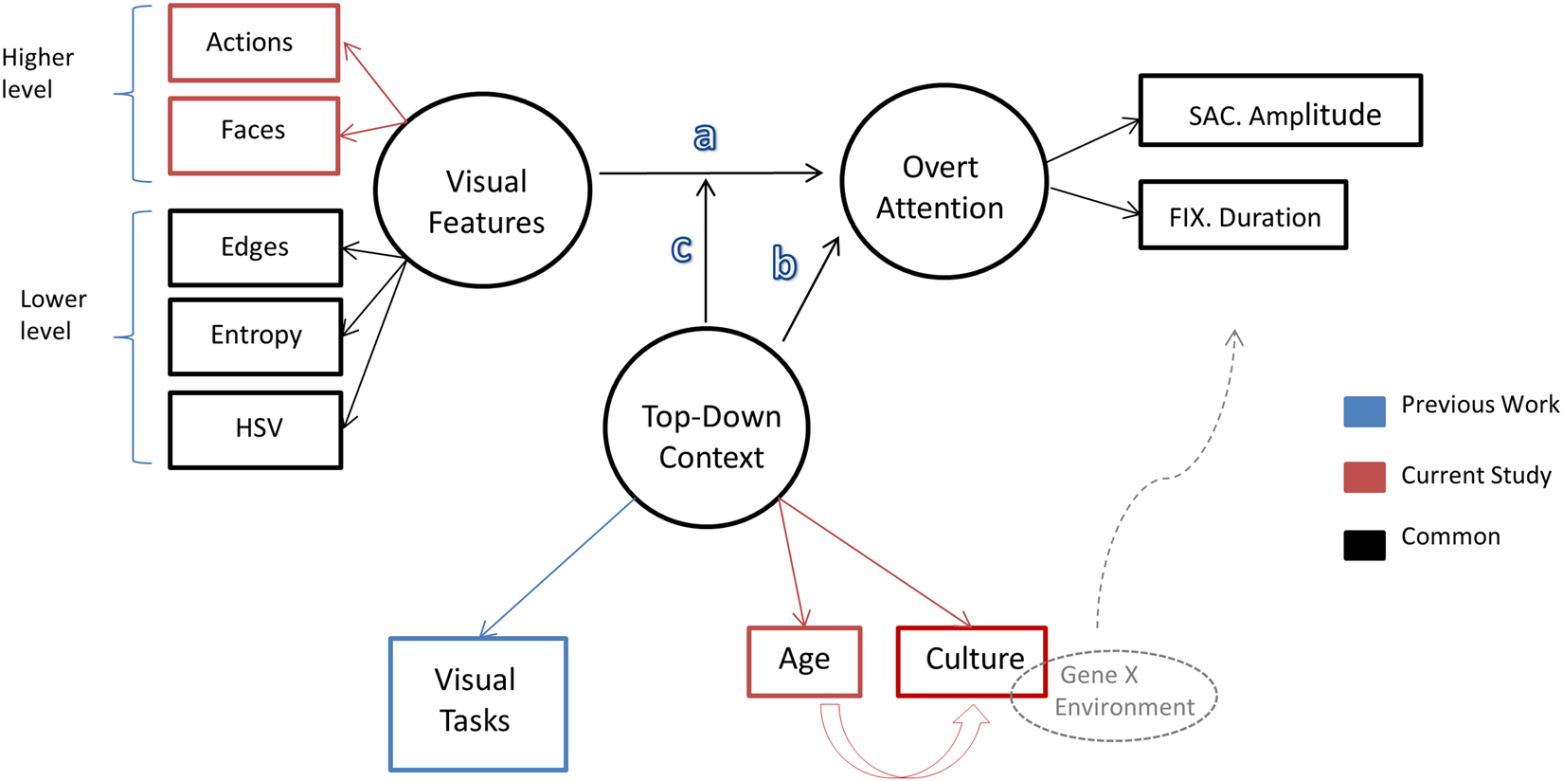
Correspondence model of the relationship between the visual environment, eye-movements, and the top-down context of the viewer (adopted and altered from)^18^. Observed variables are in rectangles, while latent variables are in circles. In this model, path ‘a’ depicts the effect of visual saliency of the stimulus on controlling eye-gaze behavior, path ‘b’ shows the non-stimulus dependent variables (age and culture in this study) that modulate eye-gaze behavior, and path ‘c’ shows a possible moderation of the degree to which visual features guide eye-movements by the cultural background and developmental stage of the viewer. HSV is Hue, Saturation, and Value variables which are simple descriptors of the color content. SAC is saccade and FIX is fixation.

In examining differences in overt visual attention patterns we used a modified version of the model by Kardan *et al*.^8^, which proposed a model for overt attention that included how the features of visual stimuli and the “context of the viewing” (as indexed by the goals of the viewers) interact to alter the spatial and temporal properties of saccadic eye-movements. By incorporating culture and age as other contexts of viewing (Fig. 1) we altered the model to be used in this study. In this model, path ‘a’ depicts the effect of visual saliency of the stimulus on controlling eye-gaze behavior^18, 31–34^. Alterations were made in the visual features of the model to include higher level features that are of interest in current study’s stimuli. Specifically, actions and faces in the background or the foreground (far or close to the camera) are added to the visual features. As such, the active foreground ROI is a region with high value for faces and actions (happening close to the camera), the active background is a region with medium value for faces and actions (happening far from the camera), and passive background is devoid of faces and actions. Path ‘b’ shows the non-stimulus dependent variables (age and culture in this study) that modulate eye-gaze behavior. Path ‘c’ shows a possible moderation of the degree to which visual features guide eye-movements by the cultural background and developmental stage of the viewer.

In addition to the importance of assessing age-dependent cultural bifurcations in attention patterns for our study, nonculture-dependent effects of development on eye-movement behavior could also have important implications. In terms of age differences, there is little previous literature directly linking development after early infancy to overt visual attention^35, 36^. In adults it is known that saccade planning and execution of overt attention (i.e., involving an actual eye movement) involves the posterior cortex and frontal cortex whose inhibitory capabilities increase throughout development^37, 38^. Switching one’s focus of attention from active foreground to active background and also to the static background requires disengagement of attention from the higher salient region of interest (active foreground). Thus, we hypothesized that older participants would fixate less to the active foreground (because they can more easily disengage from it), while younger participants would fixate more to the active foreground and thereby miss information from the other ROIs.

In summary, this paper set out to achieve two goals. The first goal was to identify cultural differences in overt attention deployment at the level of saccadic control in conjunction with the visual features of environments. The second goal was to identify age related changes in the deployment of overt attention over development. Both of these goals were examined utilizing a comprehensive model of overt attention deployment (see Fig. 1).

## Results

There are three analyses in this study whose results are presented in this section. The first two analyses are more traditional univariate analyses. The first one is to test the hypotheses we inferred from the works on analytic vs. holistic cognitive styles, as well as research on third-party learning about what visual contents are likely to be of higher priority for Mayans compared to US participants. The second analysis is to test the ‘hummingbird’ eye-movement pattern to see whether Mayans shift their fixations more often/rapidly. The third analysis is a more comprehensive multivariate analysis directly related to the theoretical model proposed in Fig. 1.

### Cultural and developmental differences in attending to different ROIs

**Figure 2:**
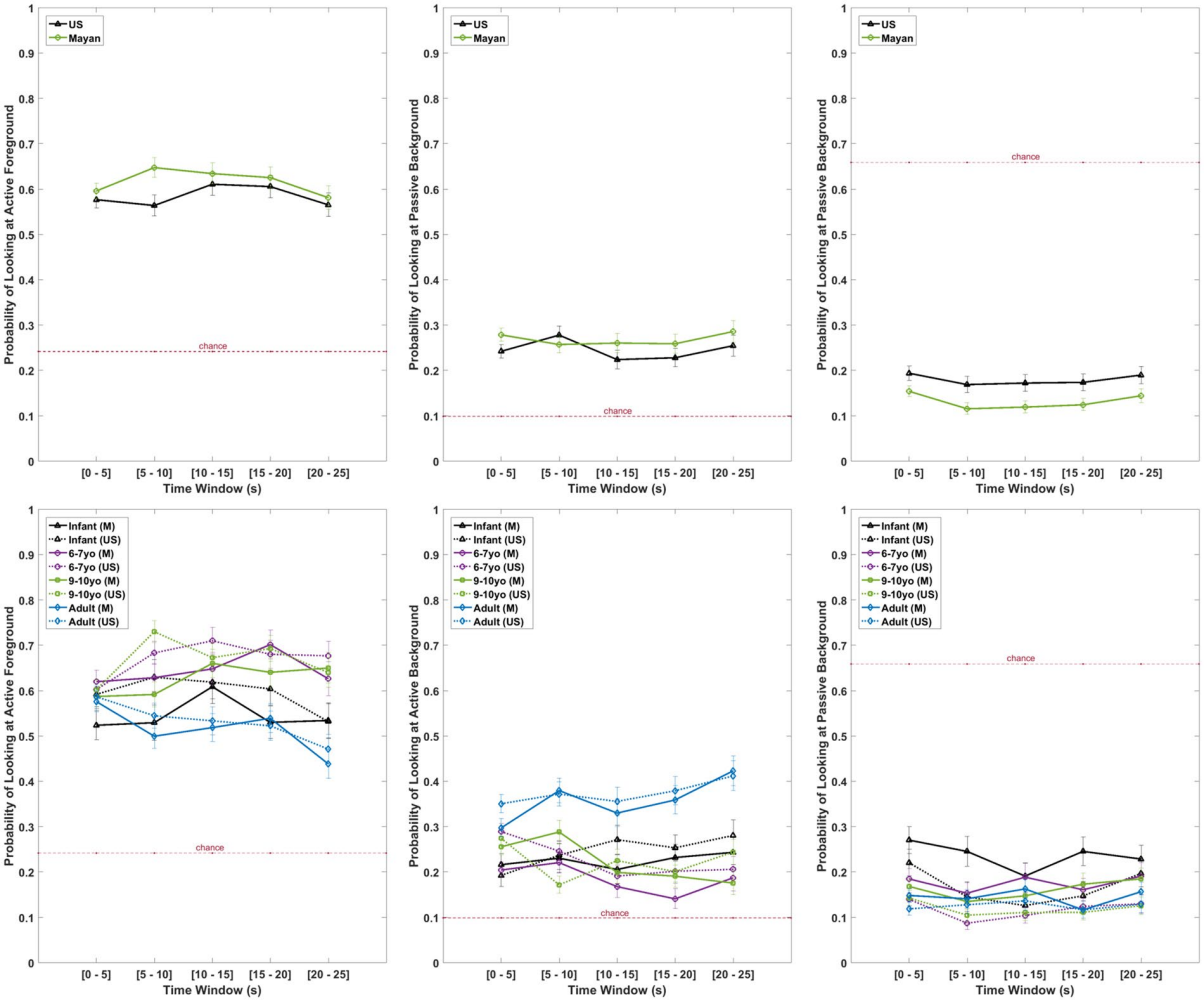
The probability of participants looking at different Regions of Interest (from left to right: Active Foreground, Active Background, and Passive Background) over time for the US participants and Mayans (top) as well as different age groups (bottom). Error bars show ± SE intervals. The red dash line shows chance as indicated by the average portion of pixels of video-screen occupied by the specific ROI.

In this section we present the results for testing our hypothesis that the probability of looking at the three ROI types (active foreground, active background, passive background) changes based on the culture and age of the viewer. Probability of looking is the proportion of each 5-second window in the video (time window) spent looking at a particular ROI, as indicated by the sum of the fixation durations in the 5-second window that that fixation’s coordinates falls within the borders of that ROI. The probabilities are then adjusted to the ROI size within each video. Table 1 shows the results of a linear mixed model fit by maximum likelihood for the effects of culture and age on the probability of looking at the 3 ROI’s with random intercepts for subjects and videos. As can be seen from the table the main effect of ROI is significant. This is not surprising and supports part of the pathway ‘a’ in Fig. 1 related to allocation of attention to the higher-level visual features with higher salience. This main effect of ROI can be seen in Fig. 2 where probability of looking is well above chance (i. e., predicted probability by sheer number of pixels) for active ROIs and well below chance for passive background for all groups including infants.

**Table 1:** Results of linear mixed model predicting probability of attending to different ROI’s. Probabilities are adjusted to ROI sizes.

Source	Df	Sum Sq	Mean Sq	F value	P value
Culture	1	1.1	1.1	0.26	0.6132
**ROI**	2	9543.4	4771.7	1069.12	0.0001*
Age	3	317.4	105.8	23.71	0.0001*
Time	4	25.8	6.4	1.44	0.2169
**ROI** * **Culture**	2	104.8	52.4	11.75	0.0001*
Culture * Age	3	6.7	2.2	0.5	0.6831
**ROI** * **Age**	6	1326.5	221.1	49.54	0.0001*
Culture * Time	4	16.5	4.1	0.93	0.4473
**ROI** * **Time**	8	76.2	9.5	2.13	0.0295*
Age * Time	12	91.4	7.6	1.71	0.0587
**Culture** * **ROI** * **Age**	6	60.7	10.1	2.27	0.0345*
Culture * ROI * Time	8	52.7	6.6	1.48	0.1602
Culture * Age * Time	12	28.6	2.4	0.53	0.8941
**ROI** * **Age** * **Time**	24	260.2	10.8	2.43	0.0001*
Culture * ROI * Age * Time	24	132.2	5.5	1.23	0.1977

Note: Time is time window, *Shows p < 0.05; Number of observations = 11160. Groups: Subjects = 94, Videos = 8, Residuals: df = 11037, variance = 0.5763. Model: Adj. Probability ~ Time * ROI * Culture * Age + (1|Subject) + (1|Video). Null Model: Adj. Probability ~ (1|Subject) + (1|Video). Model’s goodness of fit compared to the null model: ΔBIC = 1288, χ^2^ (119) = ^2^397.7, p < 0.0001.

More importantly, however, the probability of looking at a particular ROI type significantly varies as a function of culture (interaction between ROI type and culture), as well as age group (interaction between ROI type and age). This is in support of part of the moderation pathway ‘c’ (excluding lower-level features). Contrasts in this interaction are further scrutinized in Fig. 2.

Figure 2 shows the probability of participants looking at different ROIs over the time course of the videos for the US participants and Mayan participants (top row). Please notice that the omnibus culture * ROI * age interaction is significant, so we have included culture * age cell contrasts for each ROI as well (bottom row) Mayans were more likely to attend to the active foreground compared to participants from the US overall (Fig. 2 top left, t = 2.984, p_adjusted_ = 0.0087). US participants, however, were more likely to attend to the passive background during the viewing (Fig. 2 top right, t = 6.02, p_adjusted_ < 0.0001). We found no significant differences overall in viewing the active background between Mayans and US participants (Fig. 2 top middle, t = 0.314, p = 0.7533, N.S.).

With regards to age differences, adults attended more to the active background (Fig. 2 bottom middle) than infants (t = 10.516, p_adjusted_ < 0.0001), 6–7 years old children (t = 13.185, p_adjusted_ < 0.0001), and 9–10 years old children (t = 11.717, p_adjusted_ < 0.0001) for both cultural groups. This follows our hypothesis about less developed inhibitory control capabilities in children resulting in difficulties disengaging from the active foreground. However, this pattern is not observed when comparing infants, younger, and older children with each other.

### Cultural and developmental differences in the dynamics of eye-movements

**Figure 3:**
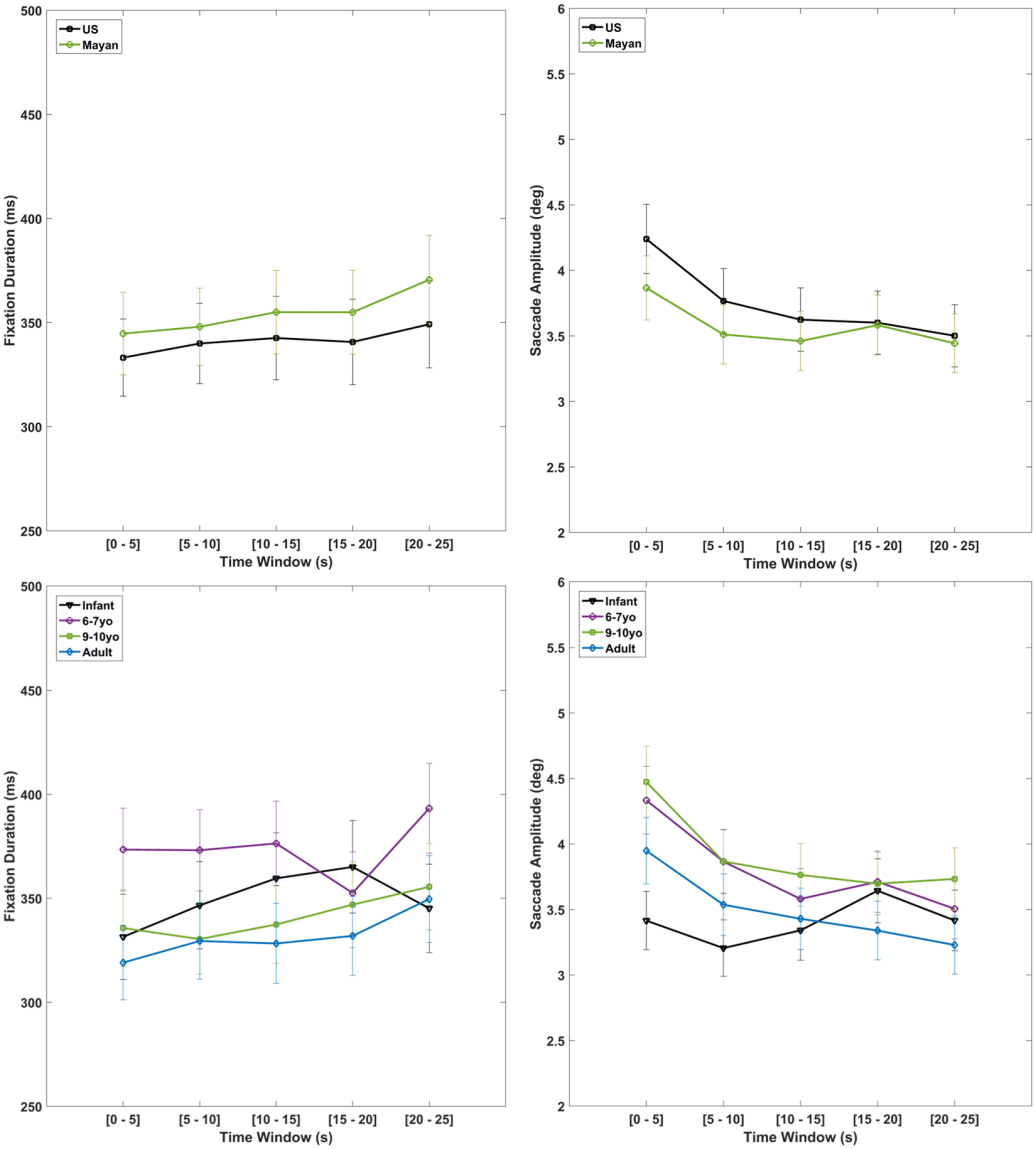
Comparison of fixation durations (left) and saccade amplitudes (right) between age groups (bottom) and culture groups (top). Errorbars show ± SE intervals.

Next, we examined how spatial (saccade amplitude) and temporal (fixation durations) dynamics of eye-movements differ between age and culture groups. Table 2 shows the results of a linear mixed model fit by maximum likelihood for the effects of culture and age on fixation durations when watching the videos. We did not observe the expected pattern of shorter fixation durations for Mayans and longer fixations for participants from the US as hypothesized by the hummingbird hypothesis^39^. As can be seen from Table 2, fixation durations for Mayan participants are not found to be significantly different from US participants (on average 361 ms for US vs. 371 ms for Mayans, see Fig. 3 top left).

**Table 2:** Results of linear mixed model predicting fixation durations.

Source	Df	Sum Sq	Mean Sq	F value	p
**Time**	4	3058691	764673	11.4166	0.0001*
**ROI**	2	26274264	13137132	196.1379	0.0001*
Culture	1	18452	18452	0.2755	0.5997
Age	3	470067	156689	2.3394	0.0719
Time * ROI	8	9401450	1175181	17.5455	0.0001*
Time * Culture	4	362369	90592	1.3525	0.2478
ROI * Culture	2	145896	72948	1.0891	0.3365
Time * Age	12	3015418	251285	3.7517	0.0001*
ROI * Age	6	1269649	211608	3.1593	0.0042*
Culture * Age	3	427119	142373	2.1256	0.0947
Time * ROI * Culture	8	637490	79686	1.1897	0.3006
Time * ROI * Age	24	2596054	108169	1.615	0.0290*
Time * Culture * Age	12	1931011	160918	2.4025	0.0042*
ROI * Culture * Age	6	969325	161554	2.412	0.0248*

Note: Time is time window, *Shows p < 0.05; Number of observations = 44006; Groups: Subjects = 94, Videos = 8, Residuals: df = 43907, variance = 66979.0. Model: Fixation Duration ~ Time * ROI * Culture * Age + (1|Subject) + (1|Video). Null Model: Fixation Duration ~ (1|Subject) + (1|Video). Model’s goodness of fit compared to the null model: ΔBIC = 269, χ^2^ (95) = 747.0, p < 0.0001.

When examining the results of the same model predicting saccade amplitudes in Table 3, we found a marginally significant effect of culture on saccade amplitudes, which is in the opposite direction of hummingbird hypothesis (Mayans having marginally smaller saccade amplitudes on average, 3.66 degrees, than US with 3.89 degrees, see Fig. 3 top right). Consequently, the ‘hummingbird’ flight pattern of viewing for Mayans that would result in their eyes traveling on average to more spatial distance during viewing was not supported. On the contrary, participants from the US covered significantly more spatial distance per second on average (11.1 deg/sec versus 10.1 deg/sec [Notice that this is not the speed of the saccades, but the average distance travelled per second], t = 2.415, p = 0.0177).

**Table 3:** Results of linear mixed model predicting saccade amplitudes.

Source	Df	Sum Sq	Mean Sq	F value	P value
**Time**	4	1946.21	486.55	47.8174	0.0001*
**ROI**	2	1811.96	905.98	89.0378	0.0001*
Culture	1	37.32	37.32	3.6682	0.0555
**Age**	3	161.75	53.92	5.2988	0.0012*
Time * ROI	8	840.97	105.12	10.3312	0.0001*
Time * Culture	4	141.97	35.49	3.4881	0.0075*
ROI * Culture	2	75.09	37.54	3.6896	0.0250*
Time * Age	12	760.28	63.36	6.2266	0.0001*
ROI * Age	6	617.64	102.94	10.1167	0.0001*
Culture * Age	3	59.02	19.67	1.9335	0.1217
Time * ROI * Culture	8	85.98	10.75	1.0563	0.3908
Time * ROI * Age	24	398.02	16.58	1.6299	0.0266*
Time * Culture * Age	12	188.91	15.74	1.5471	0.0996
ROI * Culture * Age	6	46.04	7.67	0.7541	0.6061

Note: Time is time window, *Shows p < 0.05; Number of observation = 44006; Groups: Subjects = 94, Videos = 8, Residuals: df = 43907, variance = 10.1752. Model: Saccade Amplitude ~ Time * ROI * Culture * Age + (1|Subject) + (1|Video). Null Model: Saccade Amplitude ~ (1|Subject) + (1|Video). Model’s goodness of fit compared to the null model: ΔBIC = 319, χ^2^ (95) = 696.89, p < 0.0001.

With regards to age, we found a marginal omnibus effect of age on fixation durations (Table 2) and a significant omnibus effect of age on the saccade amplitudes (Table 3). Figure 3 bottom shows no clear difference in the dynamics of fixation durations and saccade amplitudes across age groups, except for the first 5 seconds of viewing when non-infants seem to engage more in the primary examination of different parts of the stimuli (looking around in the video screen hence larger average saccade amplitude for them compared to infants).

### Results from the canonical correlations

Although the univariate results presented in the previous section are informative, they lack in two aspects. First, with those analyses we are unable to examine the spatial-temporal (saccade amplitudes and fixation durations combined) properties of the eye-movements simultaneously. If saccade amplitudes and fixation durations co-vary together in important and different ways based on age or culture, those differences will be missed with the univariate analyses, as those analyses are unable to detect those patterns. It is known from previous research that the covariance of spatial (saccade amplitude) and temporal (fixation durations) aspects of eye-movements are important when one tries to relate eye-movement behavior to attention^6, 8^. In our level of analysis, viewer’s attention to different content in the videos could manifest itself in the following ways: (1) mainly altering fixation durations (henceforth referred to as eye-movements pattern 1 or EP-1), (2) mainly altering saccade amplitudes (EP-2), (3) simultaneously altering the length of saccade amplitudes and the following fixations’ duration in the same direction (EP-3), or (4) simultaneously altering the length of saccade amplitudes and the following fixations’ duration in opposite directions (EP-4). Please see canonical correlation section in the methods for more details.

Second, the previous analyses lacked details regarding how different elements of the videos related to overt attention, and whether those attended to video elements changed based on the age and/or culture of the participants. Each video contained not only faces and meaningful movements (actions), but also a variety of lower-level visual properties such as brightness, colors, edges, etc. How those variables together with higher-level features, relate to eye-movement behavior differently based on the developmental and cultural differences of the viewers would inform us about the ‘c’ pathway in the gaze control model shown in Fig. 1. The ‘c’ pathway indicates how the relationship between contextual features of the videos and alterations in fixation durations and saccade amplitudes is moderated by the cultural and developmental context of the viewer. For these two reasons, we performed a more comprehensive multivariate analysis (see canonical correlations analysis in the methods) whose results will be discussed in this section.

**Figure 4:**
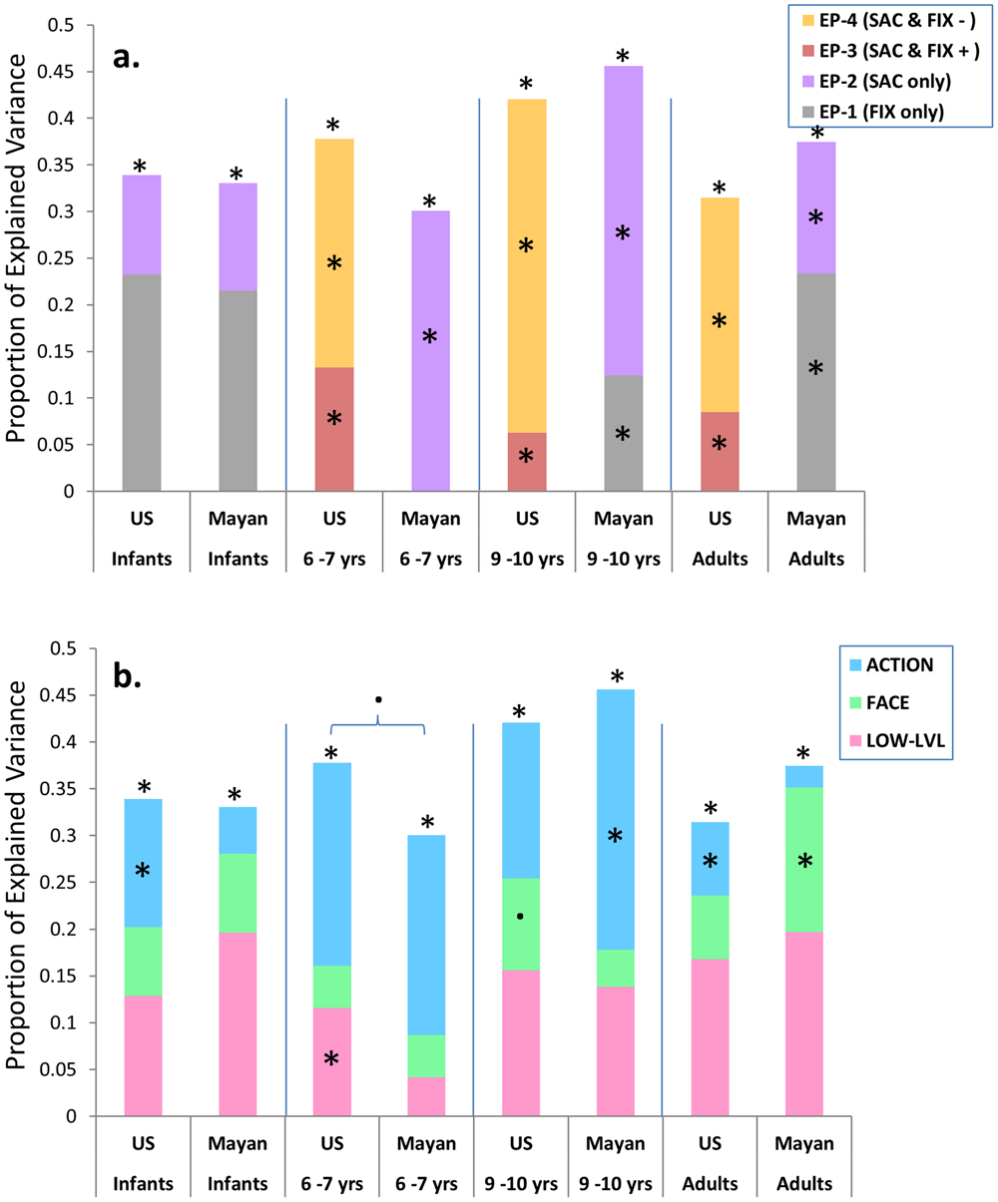
Comparison of the explained variance in the eye-movements during the view for the US participants and Mayans of different age groups. The results from the canonical correlations analyses (shown in the Supplementary Figures S1–S16) on the age-culture groups are aggregated in two ways in this figure. The variance is partitioned based on partial r^2^ from (**a**) spatial and temporal eye-movement patterns i. e., fixation durations and saccade amplitudes and (**b**) from the video-driven features i. e., actions, faces, and low level visual features (edge density, entropy, hue, saturation, and brightness). Within every age-group panel (separated by vertical lines) the * and ‘.’ inside each partition shows whether it is significantly greater than the same partition of the other culture in that age-group panel using Fisher’s r to z transformation, with p_adjusted_ < 0.05 and p_adjusted_ < 0.1 respectively. The ‘*’ on the top of a column shows that every one of the partitions in that column are significantly larger than zero. Adjustment of the p-value is done for twenty-four contrasts in (**a**) and (**b**) using the Holm-Bonferroni method.

The results from the canonical correlation analyses can be summarized in two ways. Figure 4 shows the explained variance of the visual content of the videos by the alterations to fixation durations and saccade amplitudes. While infants utilized the same eye-movement patterns (EP1 and EP2) regardless of their culture (Fig. 4a), a cultural bifurcation of US and Mayan viewers becomes obvious by the age of 6. Mayans continue utilizing the EP1 and EP2 patterns where fixation durations and saccade amplitudes are altered *independently* in response to the videos’ content, whereas participants from the US utilize EP3 and EP4 patterns where fixation durations and saccade amplitudes are altered *simultaneously* in response to the videos’ content.

The second way to summarize the results from the canonical correlation analyses is presented in Fig. 4b, where the contribution of each type of visual input (low-level visual features, faces, and actions) for explaining the variance of the viewing pattern of the US and Mayan participants in different age groups is compared. While the low-level visual features, faces, and actions are important across all age groups and cultures, the degree of their importance is to some extent culture- and also age-dependent. Specifically, infants from the US alter their eye movements in response to actions more than Mayan infants (z = 3.502, p_adjusted_ = 0.0055), US 6–7 years old children alter their eye movements in response to low-level visual features more than their Mayan peers (z = 3.224, p_adjusted_ = 0.012), and Mayan 9–10 years old children respond more to actions than their US peers (z = 3.348, p_adjusted_ = 0.0090). Finally, adult Mayans alter their eye-movements in response to faces more (z = 3.219, p_adjusted_ = 0.0115), while their US peers alter their eye-movements in response to actions more (z = 2.928, p_adjusted_ = 0.0272).

In general, our multivariate analysis shows that first, the content of the video that is modulating the eye-movements for the US participants and Mayans across different ages vary to some extent (Fig. 4b), and second that they utilize different eye-movement patterns to navigate those visual features of the videos (Fig. 4a). These findings, again, point to the pathway ‘c’ in the theoretical model in Fig. 1 as age * culture moderates how visual features guide eye-movements. Importantly, the fact that the differences between cultural groups in terms of dependence vs. independence of fixation durations and saccade amplitudes only emerges as early as 6 years of age points to the relatively rapid accumulation of different cultural experiences that results in such differences. A purely genetic account would predict the differences to be visible among toddlers as well. We will discuss this matter later in more details.

## Discussion

In this study we investigated the cultural and developmental effects on overt attention patterns of US and Yucatecan Mayan infants, children, and adults. Our findings indicate clear cultural distinctions in patterns of how attention is allocated to various visual features, as well as how the navigation of those features is carried out differently. Culture and age moderate the degree to which low- and high-level visual features of the video systematically modulate the fixation durations and amplitudes of saccades. Our results also show that US and Mayan participants utilize different eye-movement patterns to navigate the videos by the age of 6, but not as infants. Across all ages the Mayans continue utilizing separate spatial and temporal alterations in their eye-movement behavior where fixation durations and saccade amplitudes are altered *independently* in response to the videos’ content, whereas the US participants diverge and utilize patterns where fixation durations and saccade amplitudes are altered *simultaneously* in response to the videos’ content. The fact that this cultural bifurcation emerges as early as 6 years of age points to the fast accumulation of different cultural experiences that results in such differences. At the same time, lack of differences in this spatial-temporal dependency before 6 years of age support that its emergence is due to differences in cultural/environmental experience, not pure genetic differences.

Building upon the previous research on Mayans provided us with two hypotheses: one regarding ‘what’ might Mayans differentially allocate attention to, and another regarding whether that would result in the eye-movement behavior to resemble a hummingbird’s flight pattern. The results from the ROI analysis (Fig. 2) supports the prediction of the works of Rogoff and others in that Mayans will tend to the region with high people’s activity more than US participants in observational contexts. The results from the multivariate analysis (Fig. 4b) also comply with observations with regards to Mayan children learning to be more attentive to social interactions and activities even when they are not directly addressed (third-party attention hypothesis)^22, 23^.

Importantly, however, our results from analyzing the spatial and temporal properties of eye-movements (saccade amplitudes and fixation durations, Fig. 3) did not follow our second hypothesis regarding ‘how’ basic eye-movement properties might differ between groups, since participants from the US covered more saccadic distance than Mayans. We also separately analyzed the fixations only within the two active ROIs (Active foreground and Active background) because there was the possibility that Mayans were less interested in the non-person content areas (passive background) which comprises a large portion of the scenes and could have resulted in them traveling less saccadic distance than US participants. This analysis also yielded no evidence for Mayan participants traveling more spatial distance per second (10.7 deg/sec for US versus 9.6 deg/sec for Mayans, t = 2.325, p = 0.0223). Additionally, there was no evidence that US participants switch less often between ROIs than Mayans (278 mHz for US compared to 270 mHz for Mayans, i. e. once every 3.6 seconds vs. once every 3.7 seconds, respectively), which again does not support our expectations based on the ‘hummingbird flight pattern’ of eye-movements hypothesis.

These two results together imply that Mayans are indeed more attentive to passive events, but even though they might be better at noticing (or reacting to) multiple events in their visual world^12^, that attentional deployment is probably covert and not reflected in their actual overt eye-movement behavior.

Nisbett and colleagues found that US participants show more focal attention to higher salience and allocated less global attention to less salient aspects of a scene compared to cultures with less ‘analytic cognitive style’^21^. The results from our analyses examining the attention to active foreground and background, and passive background ROIs (Fig. 2) shows no evidence for Mayans being less analytic or more hollistic. This particular theory should be applied with caution, though, for a couple of reasons. First, it is unclear whether characteristics of Mayan communities (or any other culture) can be boiled down to the individualism - collectivism spectrum. Second, introducing social content to the visual stimuli could change how overt attention is deployed relative to static images with little to no social content. Moreover, Chua *et al*.^14^ examined fixations during the first 1–2 sec of exposure to a static stimulus scene composed of a focal object and its relatively impoverished context. This procedural difference is another discrepancy between the studies.

A bifurcation between US and Mayan participants was found in eye-movement behavior by the age of 6. It is known that fixation durations reflect processing time and saccade amplitudes reflect attentional breadth, but how do alterations in fixation durations and saccade amplitudes in response to the visual content of a video relate to attentional salience? One way to think about this is that if longer fixations are consistently made on certain parts of a scene or video, those regions are thought to be receiving more attention from the viewer. If some of those regions have consistent content across most of the viewers, we can think of that visual content as being important for visual attention for that group of viewers. A similar argument could be made when considering saccade amplitudes. If longer saccades are consistently made during the viewing to center the fovea on certain parts of a scene or video, those regions seem to be more attention-grabbing because the viewer is willing to rush through and ignore a larger portion of the scene to fixate on those salient areas. Again, if this is systematic across many viewers we would call the content of those areas as being *important* for visual attention for that group of viewers. While some research on static scenes/texts suggests that two relatively independent mechanisms are involved in controlling saccades and fixations for adults^40–42^, there are others that posit a high dependence between these spatial and temporal aspects of eye-movement behavior^43^. Our results provide evidence that whether these eye-movement characteristics vary independently or co-vary together depends on the cultural experience of the observer. ^[^For concerns about the sample size in each age-culture cell see supplementary section ‘Reproducibility of the canonical correlation models’].

In terms of our gaze control model (Fig. 1), we reduced the videos into five lower-level visual features (local hue, saturation, brightness, entropy, and edge density at fixations), as well as four regions of interest indicating faces or actions in the background or in the foreground. We then related the spatial and temporal properties of the eye-movements separately in each age-culture group to the video-driven variables in a canonical correlation analysis. We found that culture and age variations in the viewer moderated the relationship between video-driven features and the eye-movements (pathway ‘c’ in Fig. 1), as the extent to which each video-driven feature related to alterations in temporal and spatial aspects of eye-movements interacts with both age and culture. More specifically, our multivariate analysis shows that first, the content of the video that is modulating the eye-movements for the US participants and Mayans across different ages vary to some extent (Fig. 4b), and second that they utilize different eye-movement patterns to navigate those visual features of the videos (Fig. 4a), suggesting that age * culture moderates how visual features guide eye-movements. This model expands on the previously proposed model for influences on visual overt attention in two ways. First, we introduced both developmental and cultural variation in the observers, making it possible to model these less thoroughly investigated top-down aspects of overt attention. Second, the visual stimulus in this study was dynamic (i. e., videos) and contained social information, which enabled us to obtain a more comprehensive and realistic model for stimulus-dependency of overt attention by introducing action/movement and human faces to previous model with static scenes^8^.

There are several cultural connotations that could result in differences in *what* is more salient for visual attention and hence more influential in guiding the eye-movements (Figs 2 and 4b). The observational learning among Mayan communities contrasts with the didactic system of education in westernized countries such as the US. We found that Mayans attended less to the passive background than US participants. This could stem from the fact that children are incorporated into or segregated from the adults’ world differentially between the cultures, with more participation in Mayan culture (self-organized attention that is habitually open to a range of events) and more instructive teaching (other-organized attention that is habitually focused on one reinforced event) in the US. Hence, it is crucial for Mayan children to find relevant activities among adults and maintain attention to them without being distracted by the non-active visual stimuli.

With regards to *how* the eyes travel upon the salient features, the mechanism through which the cultural bifurcation in the eye-movement patterns (Fig. 4a) between US participants and Mayans emerges by the age of 6 is unknown to us. However, we have some speculations as to the possible ‘usefulness ‘of the observed eye-movement pattern by US participants after age 3. We performed a simple computer simulation where the probability of hitting a random event at random times through a 30 second ‘viewing’ of a 1440 * 1080 pixel space when utilizing US, Mayan, or random eye-movement patterns. The results (Table S2 in the supplementary material) showed that while using the US and Mayan eye-movements was more efficient than chance to efficiently cover the ‘field of view’, the US pattern of dependent saccades and fixations was significantly more efficient than the Mayan’s. One possible reason for applying this slightly more efficient way of scanning the visual field could be because US city-dwellers are more dependent on an event being close to their foveal fixation point to actually notice it, so they have to compensate for that by searching the field more efficiently. Another explanation could be the differences in the kinds of scenes that are usually encountered in the different environments for the two communities, with Mayans being more likely to be outdoors and thus having larger visual fields. Further experiments and more realistic simulations are required to address these speculations more directly.

We found that older participants (older children and adults) make shorter fixations throughout their viewing compared to younger children and infants. Adults were also more likely to disengage attention from the active foreground region and navigate to the active background region. Previous research has shown the start of a rapid development in eye-movements behavioral control system around ages 8–12 due to improvements in effectively presetting goal-appropriate brain systems^38^. These results are in agreement with the hypothesis that saccade planning and execution of overt attention involve posterior and frontal systems^36, 37^ that change over development. In particular we think that the inhibitory capabilities of these regions with respect to gaze control might become more efficient with experience-dependent activity throughout development^44^. Consequently, a more strategic resolution of competition in the visual environment can be utilized by adult viewers by not over-attending to the most salient events, as opposed to a more winner-takes-all strategy due to non-efficient inhibition. Another explanation for this could be inferred when examining Fig. 2 top-right, which may indicate that adults understand what is happening in the active foreground more quickly (i.e., within the first 10 seconds) and therefore attend to it less afterwards.

There are some limitations to the current study that we would like to mention. First, the physical environment (in this case of a given culture) can influence one’s oculomotor behavior and/or attention style^45^. In our analysis we are not taking into account the visual environment the participants had been primed with before watching our videos. Additionally, perhaps the reason the US participants diverge from Mayans by age 6 is exposure to the modern highly urbanized physical environment of city for the long enough period of time, in which case some could argue that the bifurcation is not due to differences in cultural differences. Nevertheless, we believe that the value-system and life-style of most cultures (that have been less affected by globalization) are heavily influenced by environmental factors in ways that assessing cultural effects on the communities removed from the culture’s original environment would not be very meaningful. Second limitation that will have to be addressed in the future studies is the possibility of gene by environment effects that have delayed onsets (hence do not necessarily manifest themselves during infancy) and drive oculomotor control differences between groups (the grey dotted pathway in Fig. 1 showing potential phenotype effects). Studying people who have immigrated to the US from Mayan communities or another rural-based culture where patterns of learning and teaching are different from those of Maya with our analysis framework could be useful to tackle some of these issues.

In conclusion, our study investigated the cultural and developmental effects on overt attention patterns of US and Mayan infants, children, and adults. In addition to finding unpredicted results for patterns of attention for each cultural group, we found that when comparing the two groups there is a cultural bifurcation in utilizing dependent or independent spatial-temporal patterns of eye-movements between Mayans and the US participants after age 6, an aspect of eye-movement behavior that has previously only been studied as a universal feature. We have expanded our theoretical model of top-down and stimulus-driven effects on visual attention to include culture and age. Specifically, our results may be used for future theorizing for how culture and development influence attention to visual features in conjunction with other top-down effects such as visual task. Additionally, we showed that applying multivariate analyses in the study of eye-movement behavior helps to detect patterns that are otherwise undistinguishable with more standard univariate analyses and encourage other researchers to utilize such methods in their own research. Last but not the least, we found that children between 6–10 years old may have difficulty disengaging from overtly salient events in their visual world which could have practical implications for developing more efficient audio-visual technology and paradigms for education of children.

## Material and Methods

### Design and power

The study design is a split-plot factorial design (SPF-24.85) with culture and age as fixed between-subject factors with two and four levels, respectively. Videos and time segments within the videos were the within-subject factors (videos as a random factor with 8 levels, time as fixed factor with 5 levels). We conducted a power analysis using G * Power 3.1.9.2 software^46^ for a repeated-measures design with 2 * 4 groups and 8 * 5 measurements and found that a total sample size of 88 (11 per age-culture group) would provide us with sufficient statistical power (1 − β = 0.83) to detect small effect sizes (f = 0.1) for the between subject interaction effects (culture by age).

### Participants

**Figure 5:**
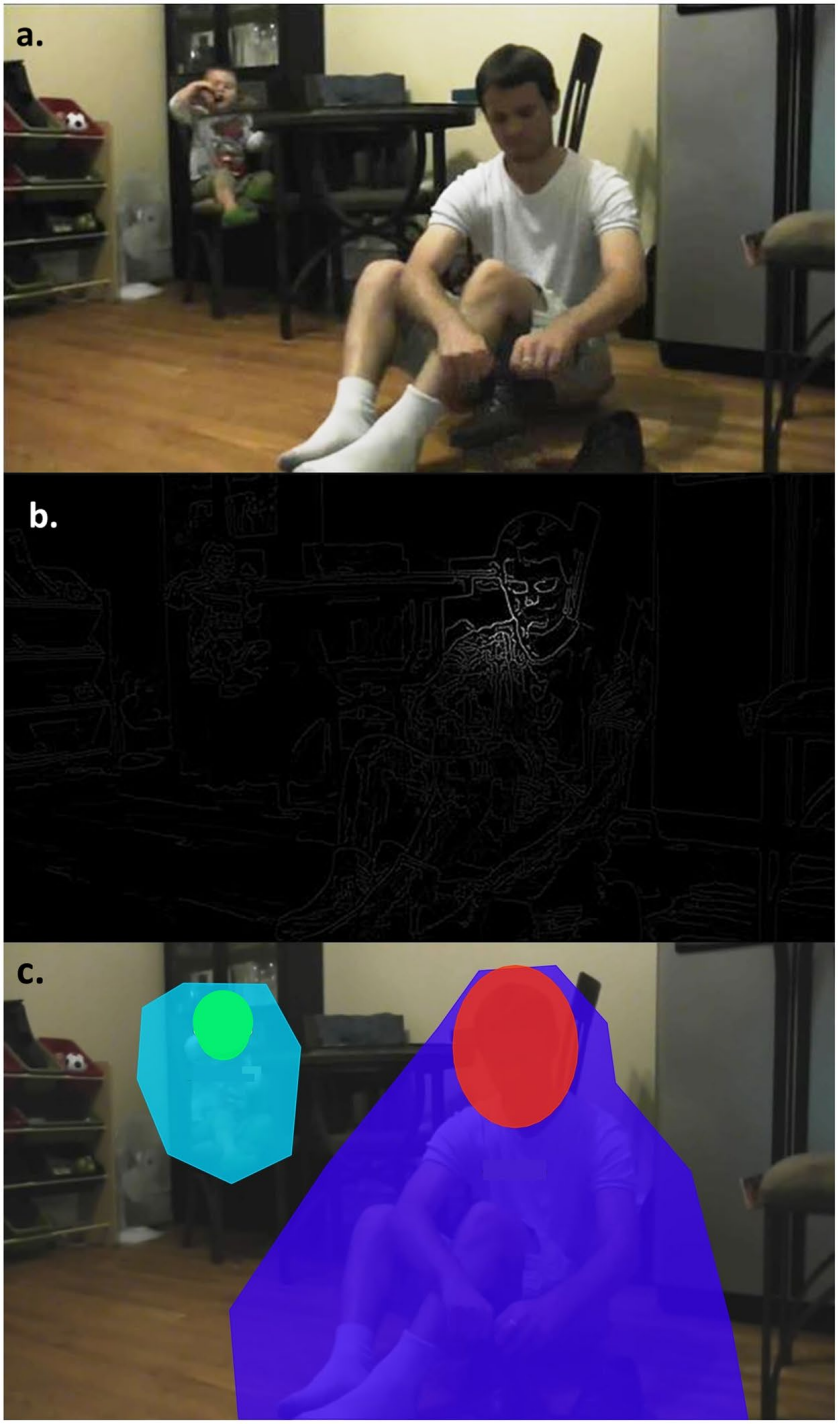
A sample frame of one of the videos (**a**), the edge density map filtered for the fixation made at that specific time in the video for one of the viewers (**b**), and the ROIs in that specific frame (**c**). Please notice that in the univariate ROI analysis Active Background consists of both background action (light blue) and background face (green), and Active Foreground consists of both foreground action (dark blue) and foreground face (red). The Passive Background consists of everything else in the frame (no color overlay).

Ninety eight participants originally participated in our study, of which 94 participants had sufficient eye-tracking data for analysis (47 Yucatec Mayan, 47 US). [To make sure the analyzed data comes from participants who were engaged in the viewing only participants that had spent at least 20 seconds of the 30 seconds (66%) looking at the screen for every video were included in the analysis, which resulted in removing 2 US and 1 Mayan infants, and 1 US adult]. Participants were divided into one of four age groups for each sample. For the Mayan sample, the groups were as follows: infants (n = 11; *M* age = 1.62 years, range = 1.11–2.03), 6- to 7-year-old children (n = 12; *M* age = 6.68 years, range = 5.79–7.58), 9- to 10-year-old children (n = 12; *M* age = 9.98 years, range = 9.08–11.56), and adults (n = 12; *M* age = 33.41 years, range = 20.95–42.19). For the US sample, the groups were as follows: infants (n = 12; *M* age = 1.61 years, range = 1.25–2.11; 2 not reported), 6- to 7-year-old children (n = 12; *M* age = 6.69 years, range = 6.00–7.62), 9- to 10-year-old children (n = 12; *M* age = 9.64 years, range = 9.15–9.99), and adults (n = 11; *M* age = 39.77 years, range = 34.67–48.50; 1 non-reported). Among the US participants, 22 reported their ethnicity as Black, 13 White, 4 Asian, 1 Hispanic, 4 of multiple ethnicities and 3 non-reported. All study protocols were approved by Institutional Review Board of the University of Chicago. All methods were carried out in accordance with guidelines and regulations provided by Institutional Review Board of the University of Chicago. All participants or their guardians provided written informed consent as administered by the Institutional Review Board of the University of Chicago. The participants or their guardians whose faces are identifiable (Fig. 5a) provided written informed consent for publication of identifying image.

### Set-up and equipment

Participants from the US were tested in a large city in the United States of America (Chicago) and Mayan participants were tested in two small villages in the eastern part of the state of Yucatan, Mexico. In both locations, eye-movements were recorded using remote corneal reflection Tobii × 2 Eye Tracker (60 Hz), for its versatility and small size. All participants were informed in their native language (English or Yucatec Maya) that they would be watching a series of movies of people engaging in everyday activities. Older children and adults were seated in front of a laptop (an HP Elitebook Mobile Workstation 8570w, screen size 34.4 × 19.4 cm) equipped with a mobile Tobii × 2–60 eyetracker. Infant participants were seated on their parents’ laps in front of the laptop. The laptop was placed on a table facing a wall to minimize distractions, at approximately 60–65 cm away from the participant’s eyes resulting in 24.5 horizontal and 18.4 vertical degrees of field of view containing the 1440 * 1080 pixels resolution videos.

### Calibration

For participants in this study, we first explained the study procedure and then sat participants in front of the eye-tracking computer and explained that they would experience a 9-point calibration process where all they had to do was look at the center of the calibration point as it appeared on the screen. For infant participants, we explained this process to the parent/caregiver and then asked them to hold their infant in a firm but comfortable manner in his/her lap as the experiment was conducted. This process was rerun until all 9-points were accurately calibrated. The participants were reminded to sit very still as they watched the series of movies. The Tobii’s calibration model uses both the light and dark pupil methods to identify the most suitable model for the light conditions and the participant’s eye characteristics.

### Videos

All Participants viewed all of our 8 videos in random order, each being 30 seconds long with 1440 * 1080 pixels resolution and presented at 30 frames per second. All eight videos contained two actors (always one adult and one child) resulting a total of 16 actors, 4 Yucatec Mayan children, 4 US children, 4 Yucatec Mayan adults, and 4 US adults. The actors performed tasks that were counter-balanced as typical tasks in a Mayan village environment (such as making tortillas, washing clothes, playing a game with rocks, etc.) done by Mayan actors and those in an urban environment in the US (putting on socks, eating food, gardening, etc.) done by US actors, with one task in each video being performed in the background and one in foreground. The adult actors and the child actors were counter balanced between background and foreground. Figure 5a shows a sample of the setup in the videos. An attention-getter was presented in the center of the screen for 500ms before each video to direct participants’ attention to the middle of the screen. The overall procedure of watching all 8 videos took about 4 minutes and 23 seconds on average, 4 minutes of which was spent watching the 8 videos.

#### Video-driven features

Our low level video features included: *edge density*, *hue*, *saturation*, *brightness*, *and entropy*. These features have previously been shown to capture important features of environmental scenes in a comprehensive manner^47–51^. To quantify the value of different visual features at each fixation, we first generated the feature map of each video frame. The maps for the visual features were created from the frames of the videos using MATLAB (MATLAB and Statistics Toolbox Release 2014a, The MathWorks, Inc., Natick, Massachusetts, United States) as in ref. 47. The visual features for each fixation were then quantified by masking the feature maps using a Gaussian filter centered at the fixation coordinates as in ref. 8. As an example, Fig. 5 shows a sample frame from a video (a) and the quantified edge density of the fixation made synchronous to that frame by a viewer (b).

Our higher-level video-driven features were faces and actions. The face and action regions of interests (ROIs) in the background and foreground of the movies were digitally hand-drawn frame by frame by a research assistant who was instructed to select the smallest closed shape that fully contain the face or the moving components in the foreground or the background of the videos in each frame, but were blind to the purpose of the analysis. This resulted in 4 ROIs for the canonical correlation analysis (background action, background face, foreground action, and foreground face). In the univariate ROI analysis, Active Background ROI consisted of the background action and background face, Active Foreground ROI consisted of the foreground action and foreground face, and Passive Background ROI consisted of everything else on the screen. Figure 5c shows the ROIs of a sample video frame.

All the variables calculated for each fixation, including the video-driven variables, the fixation durations, and saccade amplitudes were averaged over 5-second time intervals across each video for each subject for the ROI univariate analysis and also for the canonical correlation analysis, but kept at the single saccade-level for the second analysis (Cultural and developmental differences in the dynamics of eye-movement). The fixations that were made out of the boundaries of the screen, as well those longer than Mean + 3 SD (i. e., longer than 1711 ms) were excluded in all analyses.

### Mixed effects analyses

For the first two sections of the results (mixed-effects models) the following models were run in R using the lme4 package’s function ‘lmer()’^52^:

$$\begin{array}{c}{\rm{Alternative}}\,{\rm{Model}}:{\rm{DV}} \sim {\rm{Time}}\ast {\rm{ROI}}\ast {\rm{Culture}}\ast {\rm{Age}}+(1|{\rm{Subject}})+(1|{\rm{Video}})\,{\rm{and}}\\ {\rm{Null}}\,{\rm{Model}}:{\rm{DV}} \sim (1|{\rm{Subject}})+(1|{\rm{Video}})\end{array}$$

where DV is the dependent variable (probability, fixation duration, or saccade amplitude). All the statistical tests on the probabilities are done on the arcsine square root transformed values as suggested by^53^. Adjustments for p-values are done using the Holm-Bonferroni method to protect experiment-wise type I error at α = 0.05.

### Canonical correlation analysis

#### Model

**Figure 6:**
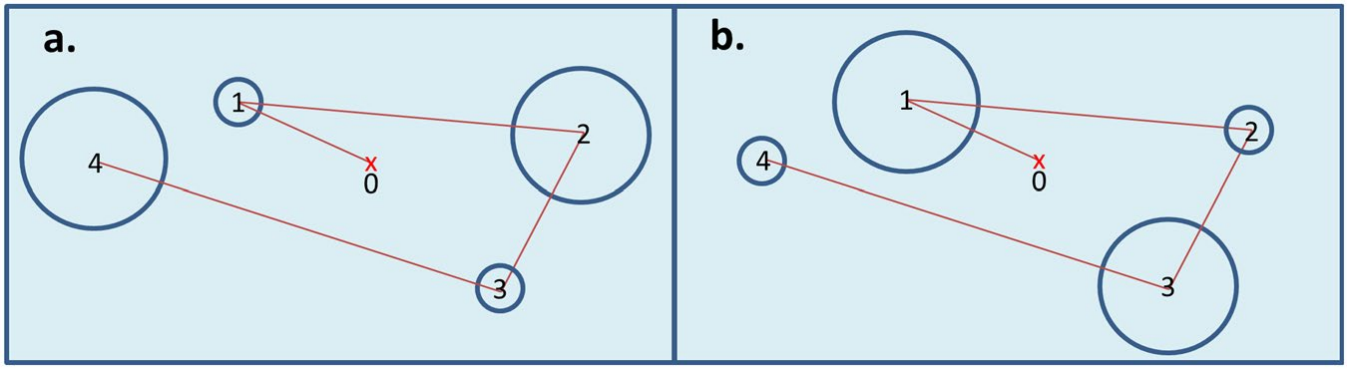
Two hypothetical eye-movement patterns over the same stimulus from two different viewers. The length of the straight red lines represent the amplitude of a saccade and the radius of a blue circle represents the duration of the fixation following that saccade.

Kardan *et al*.^8^ used a comprehensive model that distinguishes between: (1) different types of eye-movement patterns, and (2) different features of the visual environment that are attended to. For example, consider two hypothetical viewers looking at the same scene with their eye-movement trajectories depicted in the Fig. 6a and b, where the length of straight lines represents the amplitude of a saccade and the radius of a circle represents the duration of the next fixation that followed that saccade. The patterns are obviously different, but the simplest (and perhaps the most common) analysis would be comparing the mean fixation durations and mean saccade amplitudes between the two viewers, which would fail to find any differences between the viewing patterns (notice that that aggregating fixation durations or saccade amplitudes in ‘**a’** and ‘**b’** result in same values). A more nuanced analysis would be one that takes into account the covariance of the fixation durations and saccade amplitudes. For example^6^ have shown that this covariance signals part of the difference between eye-movements when performing different visual tasks. Such a multivariate analysis would be able to distinguish the patterns in Fig. 6a and b, since the fact that every long saccade is always accompanied by a long fixation in **‘a’** but by a short fixation in **‘b’**, which is discernable utilizing this multivariate approach. Using this information then makes it possible to distinguish the patterns **‘a’** from **‘b**.**’**

Therefore, we used the model in Fig. 1 which summarizes the visual features of the attended to locations of the stimulus and simultaneously takes into account both saccade amplitudes and fixation durations of the observer in this study.

#### Analysis

In a canonical correlation analysis^54, 55^, two sets of variables are related together (as opposed to many variables to a single dependent variable) and the degree of relationship between the two sets of variables is assessed. In our study, one set of variables contain the video-driven features (Set 1), consisting of low level visual features and their interactions with time of viewing (edge density, edge density * time, entropy, entropy * time, hue, hue * time, saturation, saturation * time, brightness, and brightness * time), as well as face and action ROI’s and their interaction with time (background face, background face * time, foreground face, foreground face * time, background action and background action * time). The second set of variables is the two eye-movement variables: saccade amplitudes and fixation durations (Set 2).

When performing canonical correlations, first, the weights that maximize the correlation of the two weighted sums (linear composites) of each set of variables are calculated (first canonical root). Then the first root is extracted and the weights that produce the second largest correlation between the summed scores are calculated, subject to the constraint that the next set of summed scores is orthogonal to the previous one. Each successive root will explain a *unique* additional proportion of variability in the two sets of variables. There can be as many canonical roots as the minimum number of variables in either of the two sets (hence 2 for our analysis). Therefore, we obtained two sets of canonical weights for each set of variables, and each of these two canonical roots have a canonical correlation coefficient which is the square root of the explained variability between the two weighted sums (canonical roots).

To obtain canonical weights for variables and canonical correlation coefficients relating eye-movements to video-driven features, we used the same approach as in ref. 56 for each culture-age group separately and then compared the loadings between them to look for systematic changes in the relationships between eye-movements and video-driven features based on the culture and age.

**Figure 7:**
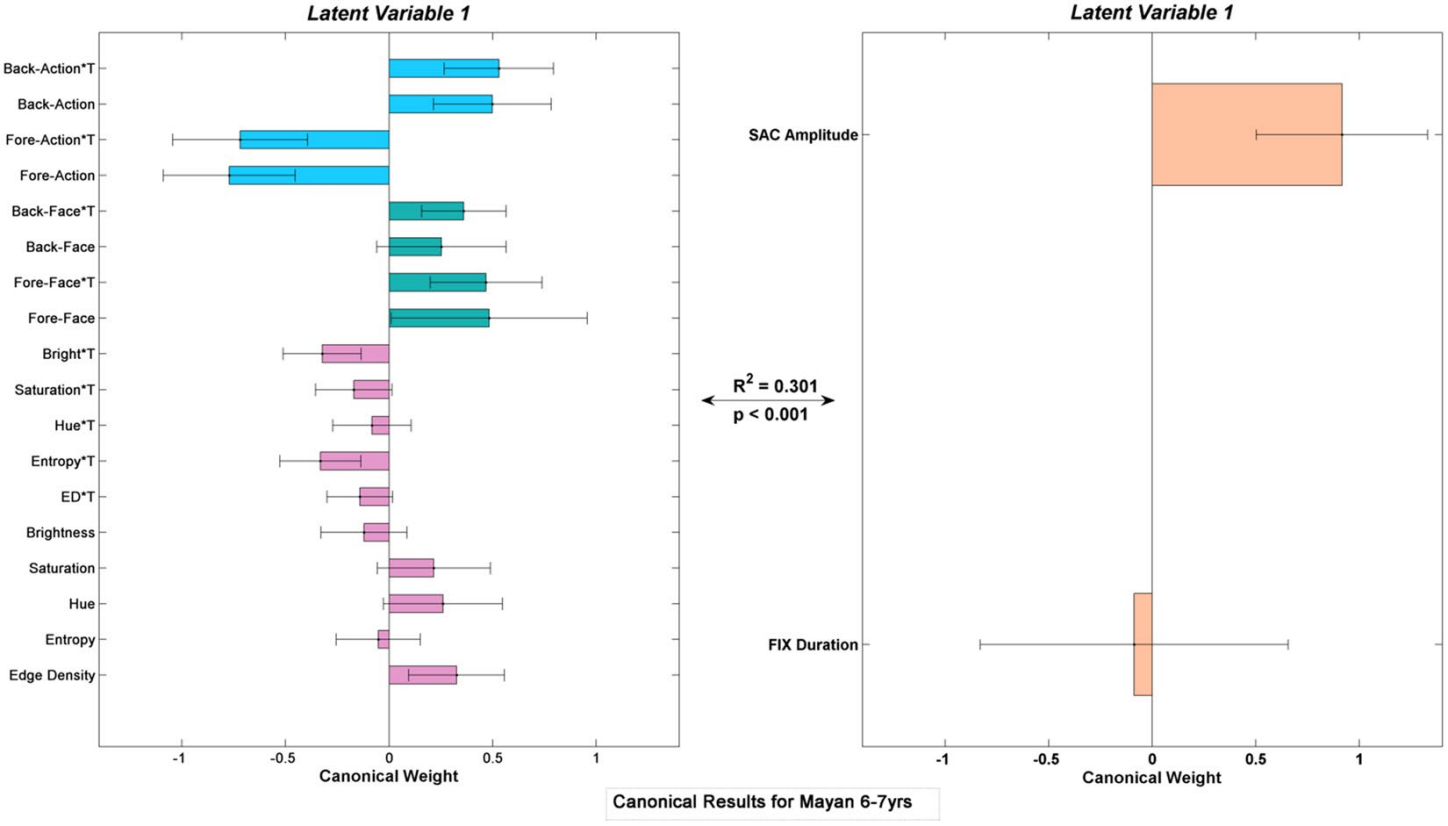
First canonical component calculated for the Mayan younger children. Left panel shows the vide-driven set of variables, where ones with the same color belong to the same group of variables (pink = low-level, green = face-related, blue = action-related). Right panel shows the eye-movement set of variables corresponding to the videos. Based on the right panel, the eye-movement pattern is an EP-2 (SAC only) in this component for this age-culture group.

We performed the canonical correlation analysis on the z-scores of the variables using MATLAB. For a more straight-forward interpretation and a better characterization of the underlying latent variables, instead of using the canonical weights, we calculated the Pearson correlation coefficients (canonical loadings) of each observed variable in the set with the weighted summed scores for each of the two linear composites. This way, each canonical root (linear composite) could be interpreted as an underlying latent variable whose degree of relationship with each of the observed variables in the set (how much the observed variable contributes to the latent variable) is reflected in the observed variable’s loading. Each variable is represented by the loading of the observed variable and its error bar, which is the 95% confidence interval calculated by bootstrapping the data (3000 samples with replacement) and choosing the symmetrical range around each average that contains 95% of all values in the loading distribution. As an example, Fig. 7 shows the first pair of latent variables for the Mayan younger children, and shows that those participants consistently make longer saccades (right side) in correspondence to higher edge density, entropy, and hue, as well as faces and actions (left side) with a large effect size (R^2^ = 0.301). There are 8 age-culture groups with two canonical components for each, resulting in 16 pairs of latent variables, which are shown in supplementary Figures S1–S16.

Since we have two eye-movement variables (fixation durations and saccade amplitudes), there are four possible eye-movement patterns that can be related to the visual content of the videos. The viewer’s attention to different content in the videos could manifest itself in the following ways: (1) mainly altering fixation durations (henceforth referred to as eye-movements pattern 1 or EP-1), (2) mainly altering saccade amplitudes (EP-2), (3) simultaneously altering the length of saccade amplitudes and the following fixations’ duration in the same direction (EP-3), or (4) simultaneously altering the length of saccade amplitudes and the following fixations’ duration in opposite directions (EP-4). For example, Fig. 6a and b showed an example of EP-3 and EP-4, respectively, and Fig. 7 right panel shows an EP-2.

After computing all the latent variable pairs, we implemented methods similar to ref. 8 to aggregate the canonical correlation results and make the results figures such as Fig. 4 in the results section. The partial r^2^ of every significant variable on the right side for every latent variable pair was calculated as the square of its loading divided by the sum of squares of all the loadings on that side. Summing the partial r^2^ of a variable across the two components then results in the proportion of covariance in eye-movements explained by that specific variable, since the components are orthogonal to each other. We then summed the partial r^2^ from each variable from the same group (pink colored for low level features, green colored for face variables, and blue colored for action variables in Fig. 7 and Figures S1–S16) to create Fig. 4b, where the partitions have the same color as their original group of variables. Another way of partitioning is based on the latent variables on the right side (the eye-movement patterns), which is simply done by identifying the pattern as an EP-1, EP-2, EP-3, or EP-4 based on the loadings on the saccade amplitude and fixation duration variables and assigning the explained variance (R^2^) to that pattern. Figure 4a is the result of this way of partitioning the shared variance of video-driven features and eye-movements.

## Electronic supplementary material

Supplementary Material
