# Supplementary material for: Cultural and Developmental Influences on Overt Visual Attention to Videos
Source: Sci Rep. 2017 Sep 12;7:11264. doi: 10.1038/s41598-017-11570-w (PMC5595807; doi:10.1038/s41598-017-11570-w)
Supplement: Supplementary file 1 — Supplementary Material [file 41598_2017_11570_MOESM1_ESM.pdf]

Supplementary material for Cultural and Developmental Influences on Overt Visual Attention to Videos

Authors: Omid Kardan[1], Laura Shneidman[2], Sheila Krogh-Jespersen[3], Suzanne Gaskins[4], Marc G. Berman[1], Amanda Woodward[1]

Affiliations:

[1]University of Chicago, Department of Psychology.

[2] Programa Interdisciplinario sobre Política y Prácticas Educativas, Centro de Investigación y Docencia Económicas

[3]DePaul University

[4]Department of Psychology. Northeastern Illinois University

Address questions to:

Omid Kardan okardan@uchicago.edu or Marc Berman bermanm@uchicago.edu or Amanda Woodward woodward@uchicago.edu

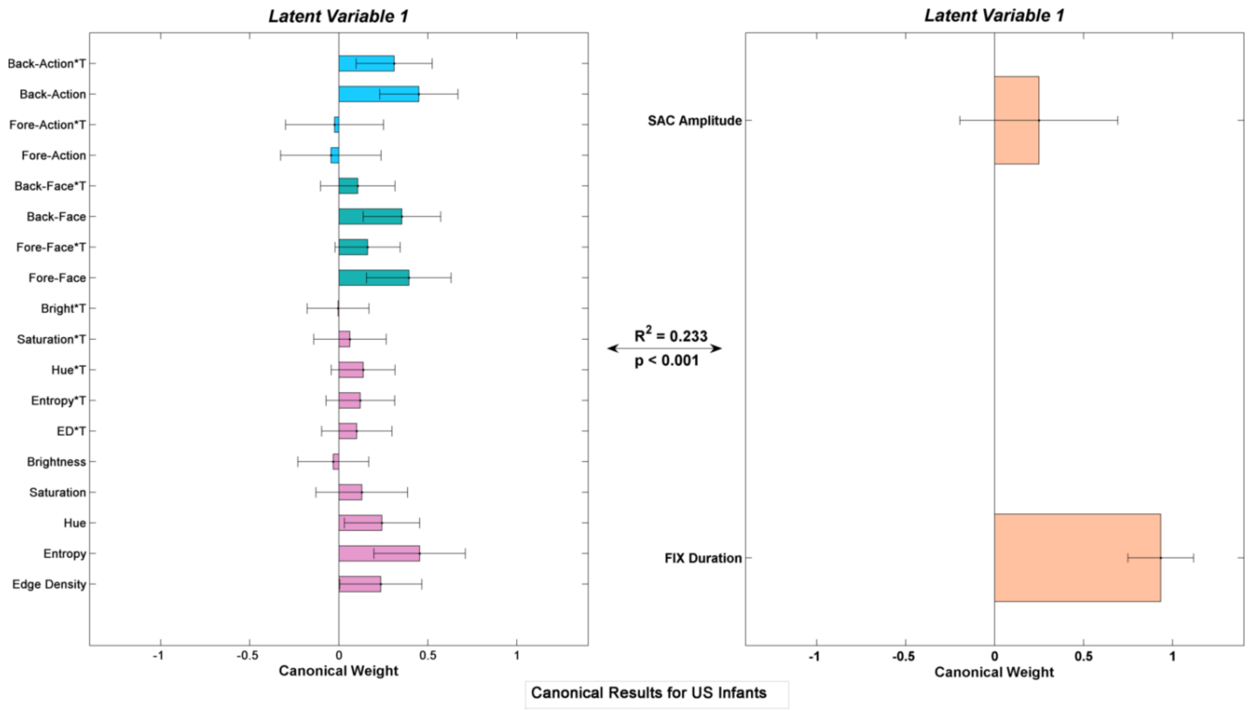

Figure S1. First component of the canonical results for the US infants.

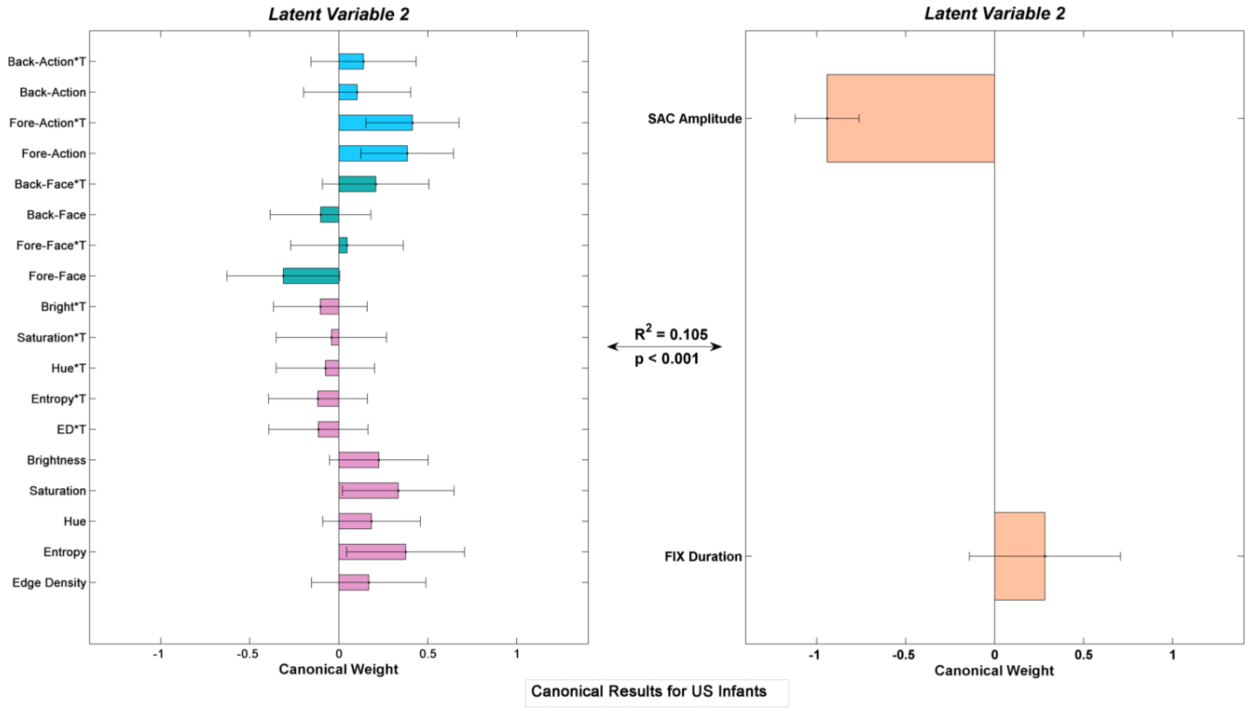

Figure S2. Second component of the canonical results for the US infants.

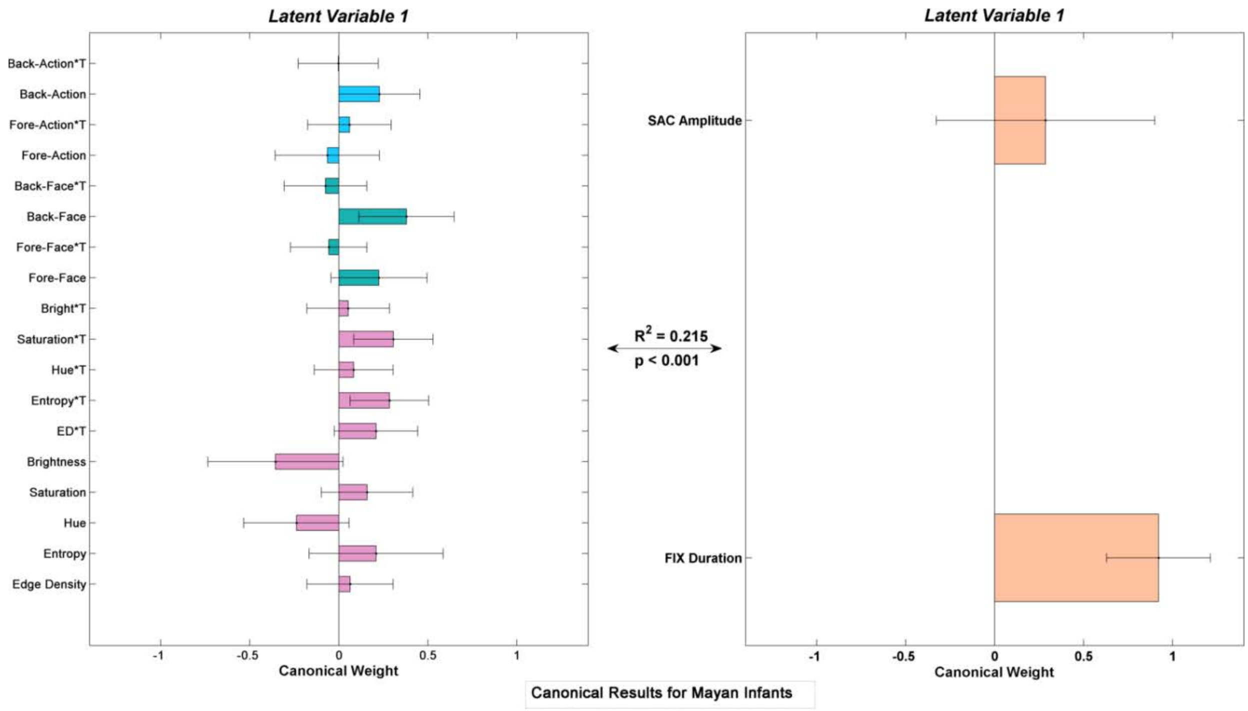

Figure S3. First component of the canonical results for Mayan infants.

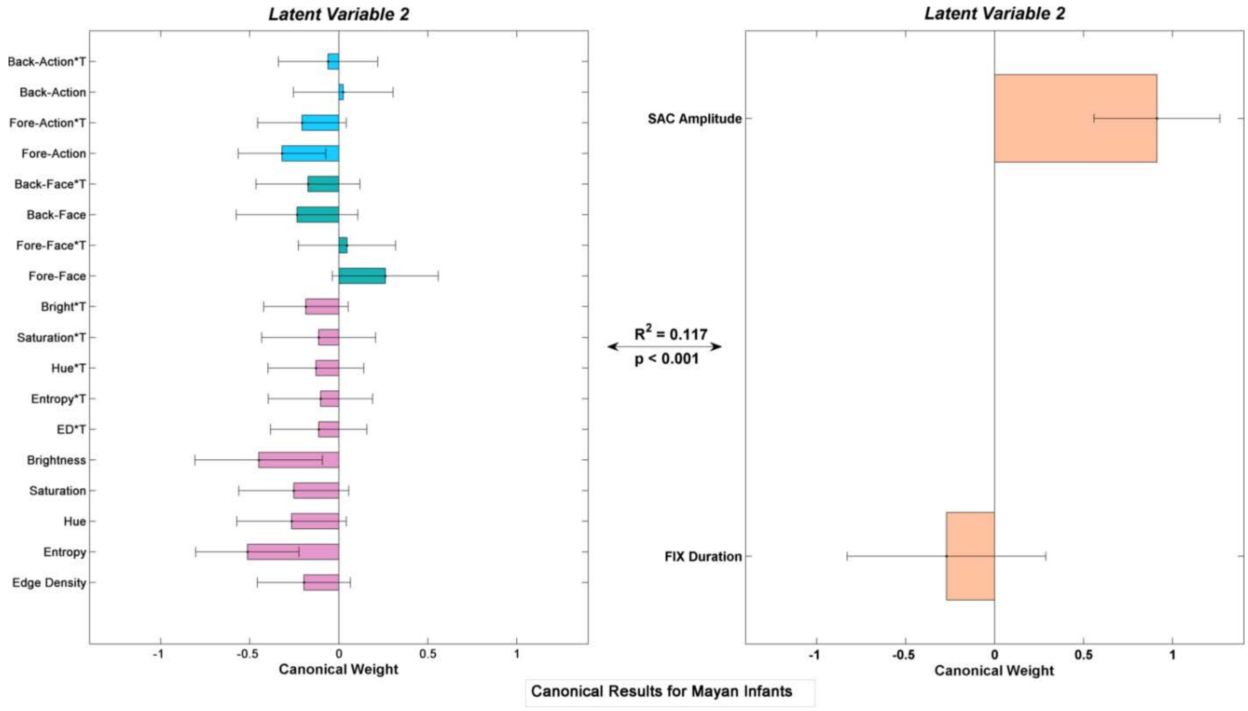

Figure S4. Second component of the canonical results for Mayan infants.

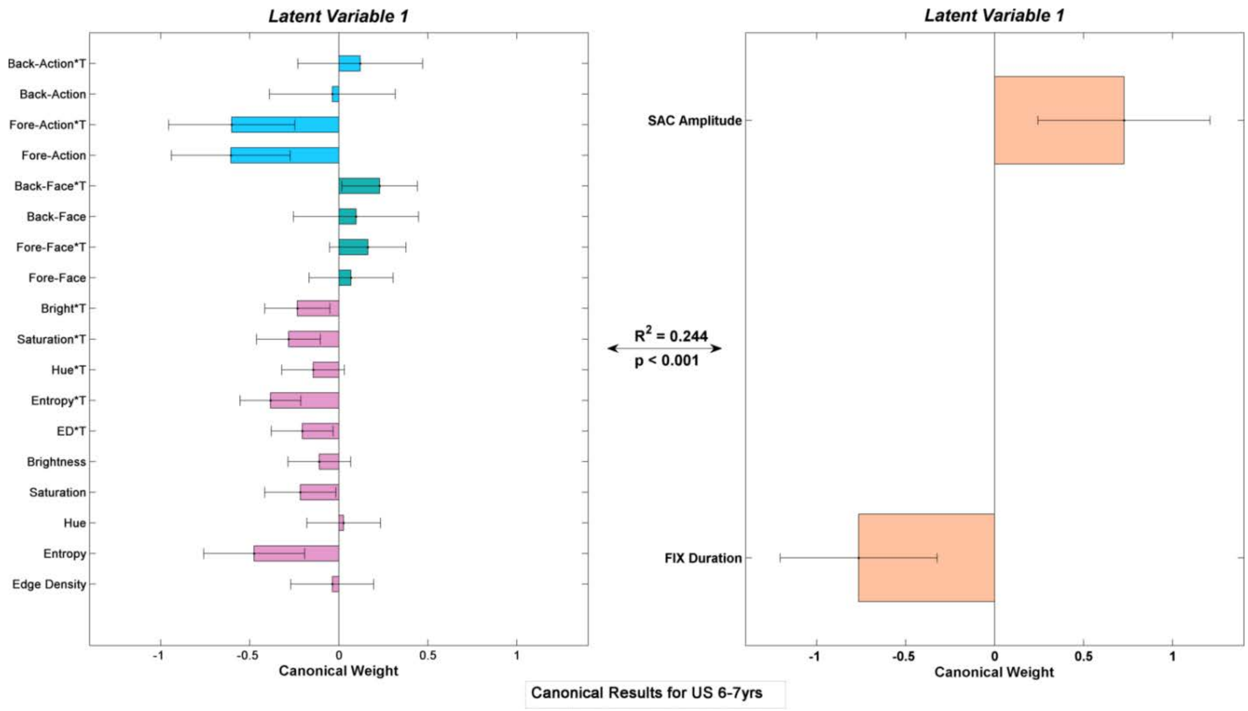

Figure S5. First component of the canonical results for the US younger children.

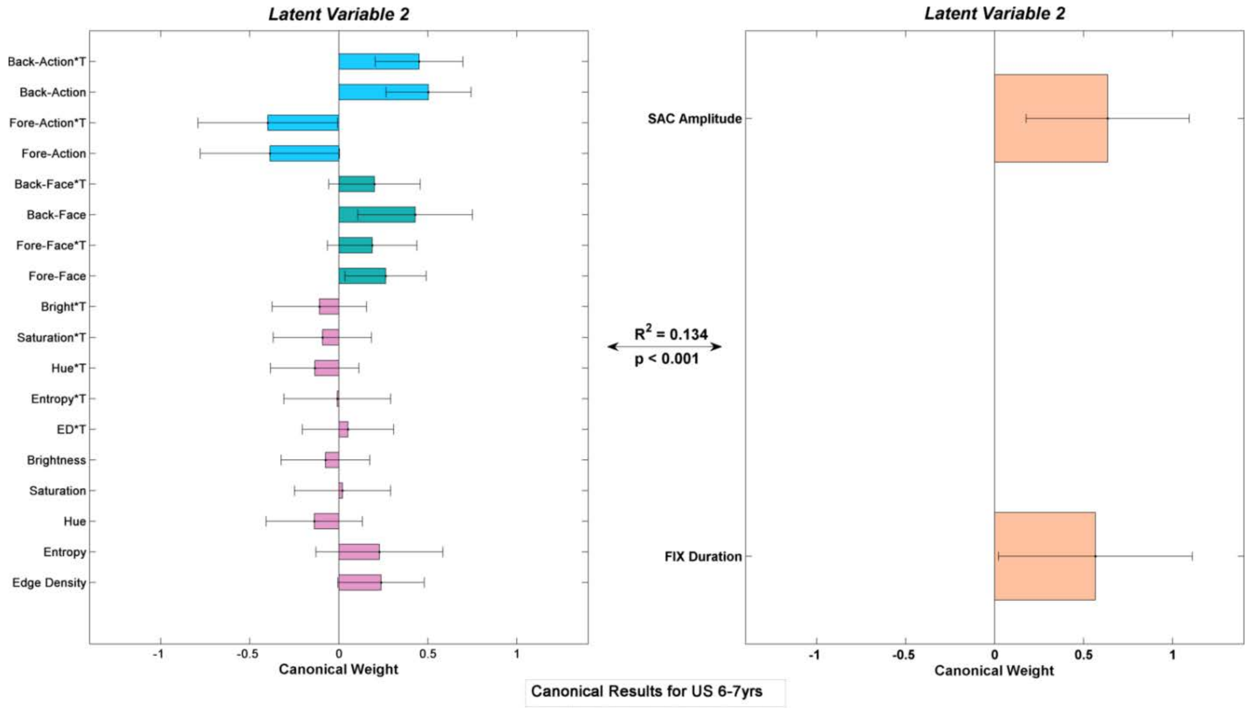

Figure S6. Second component of the canonical results for the US younger children.

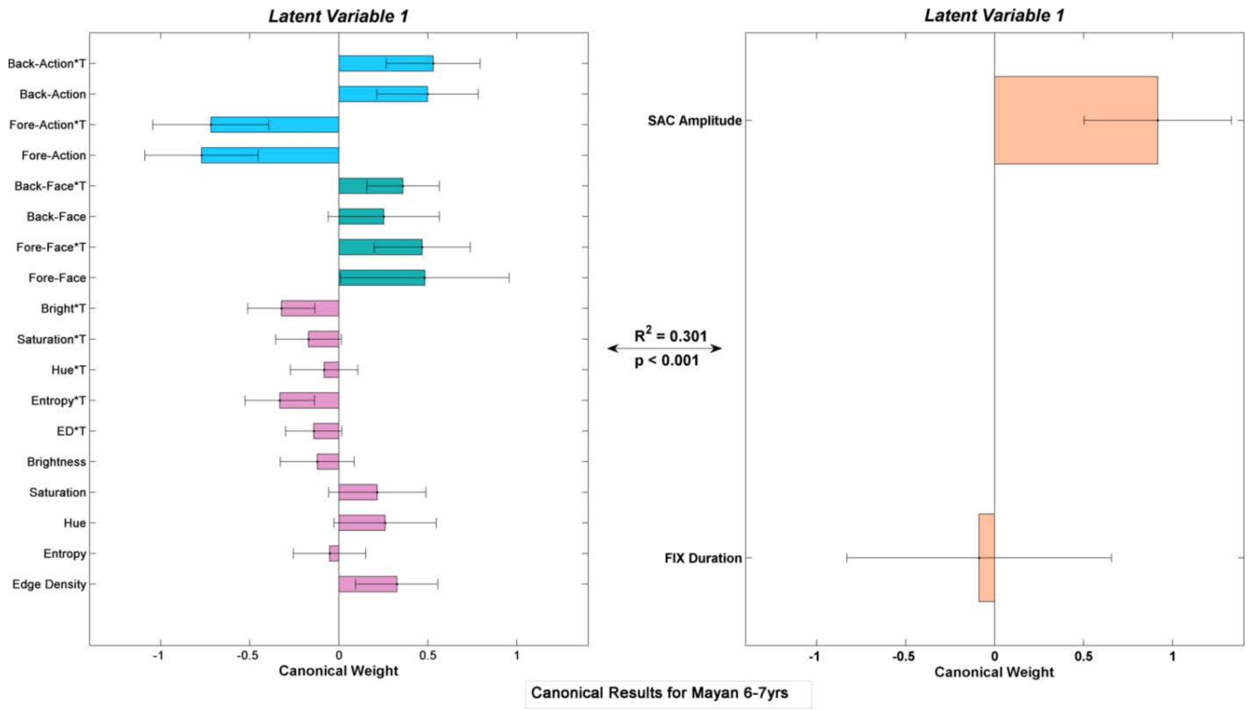

Figure S7. First component of the canonical results for Mayan younger children.

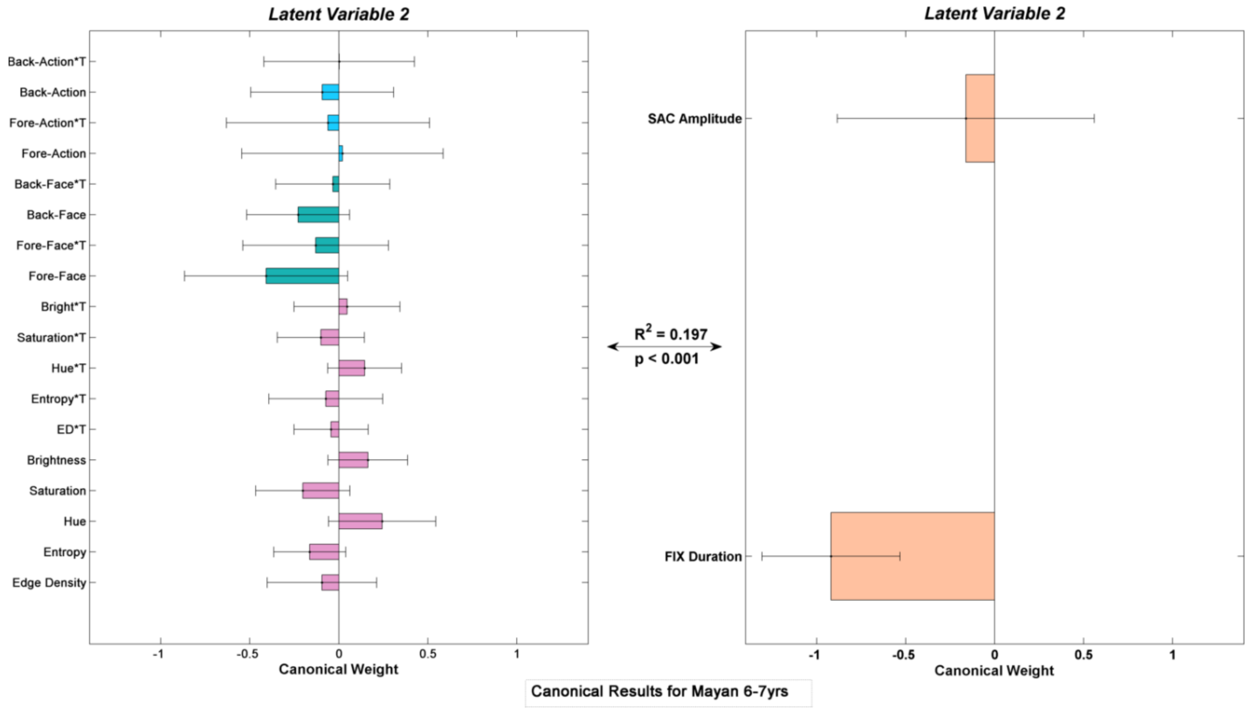

Figure S8. Second component of the canonical results for Mayan younger children.

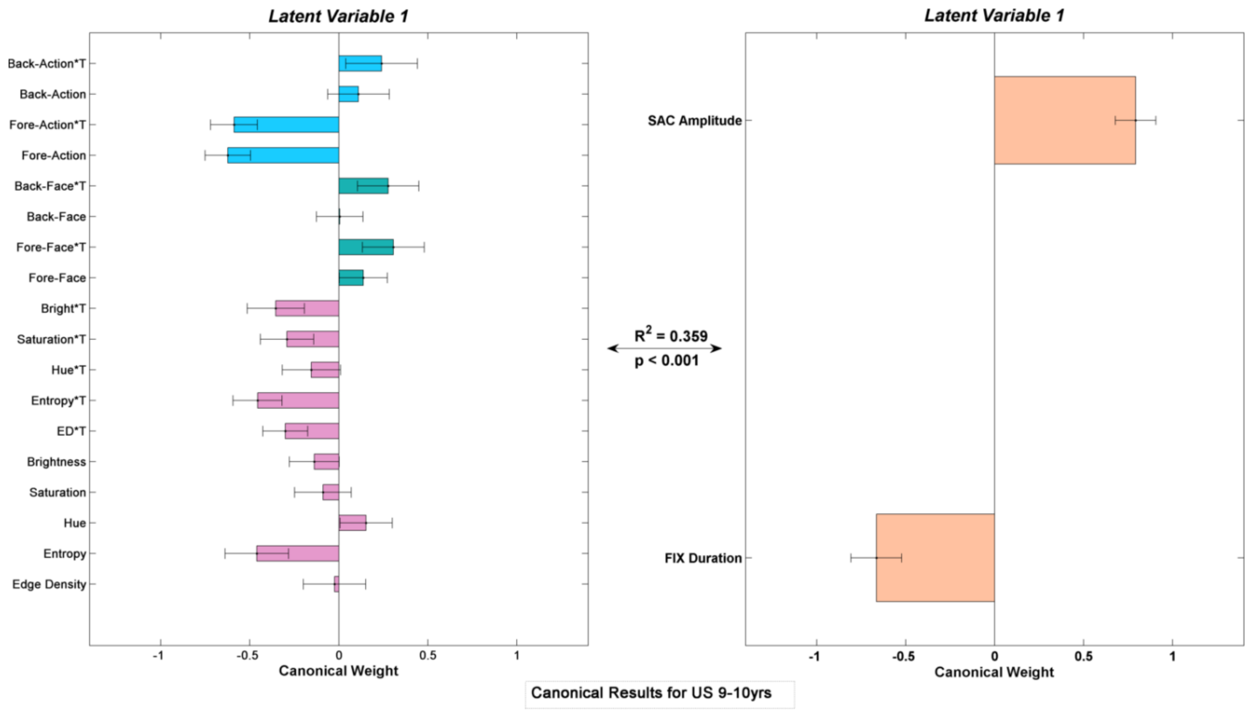

Figure S9. First component of the canonical results for the US older children.

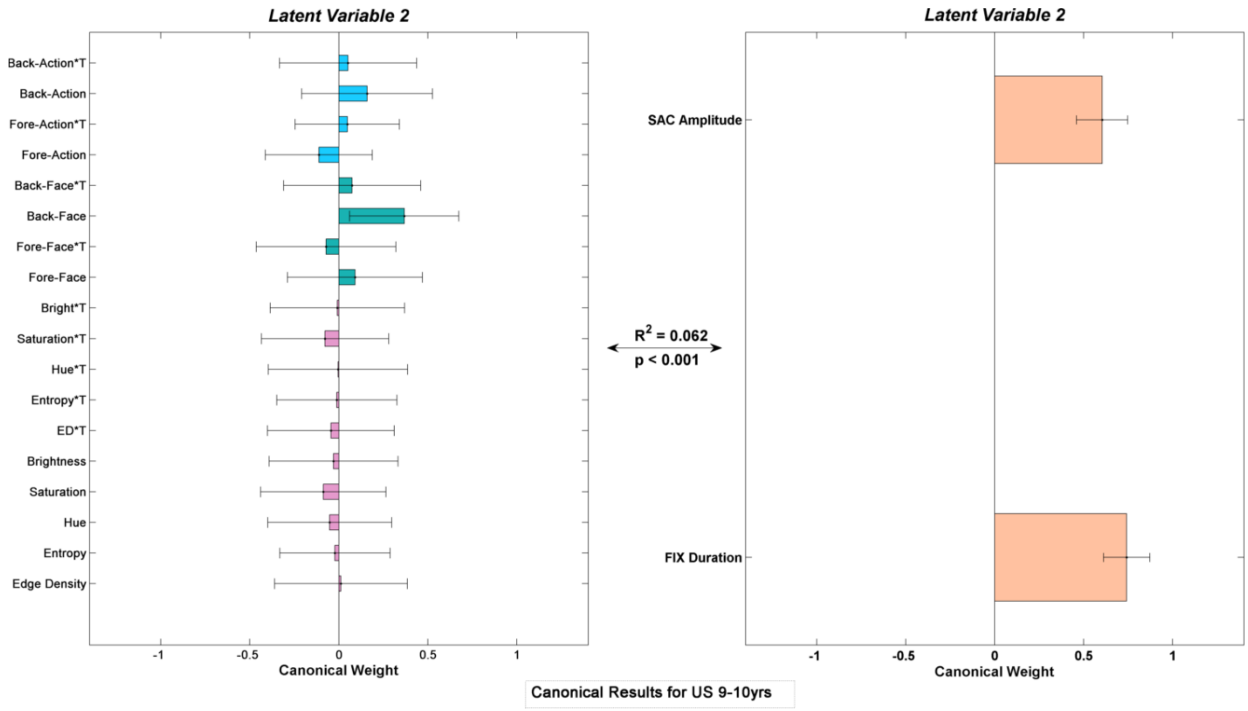

Figure S10. Second component of the canonical results for the US older children.

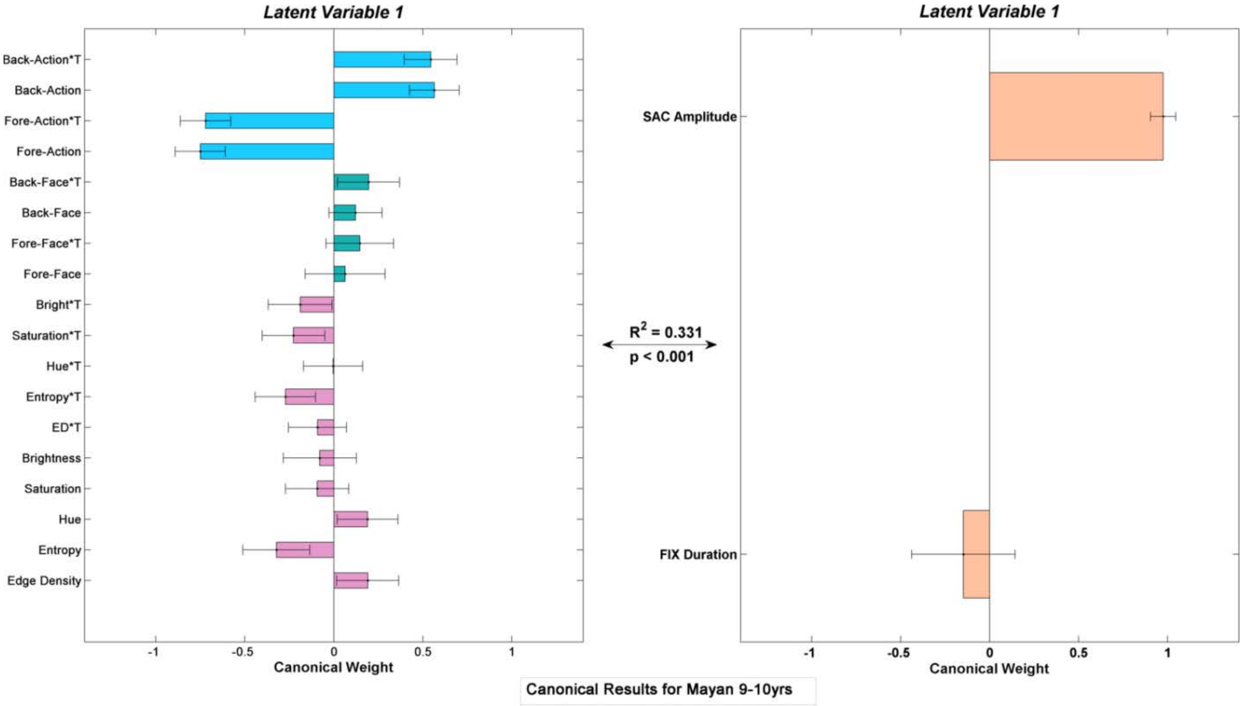

Figure S11. First component of the canonical results for Mayan older children.

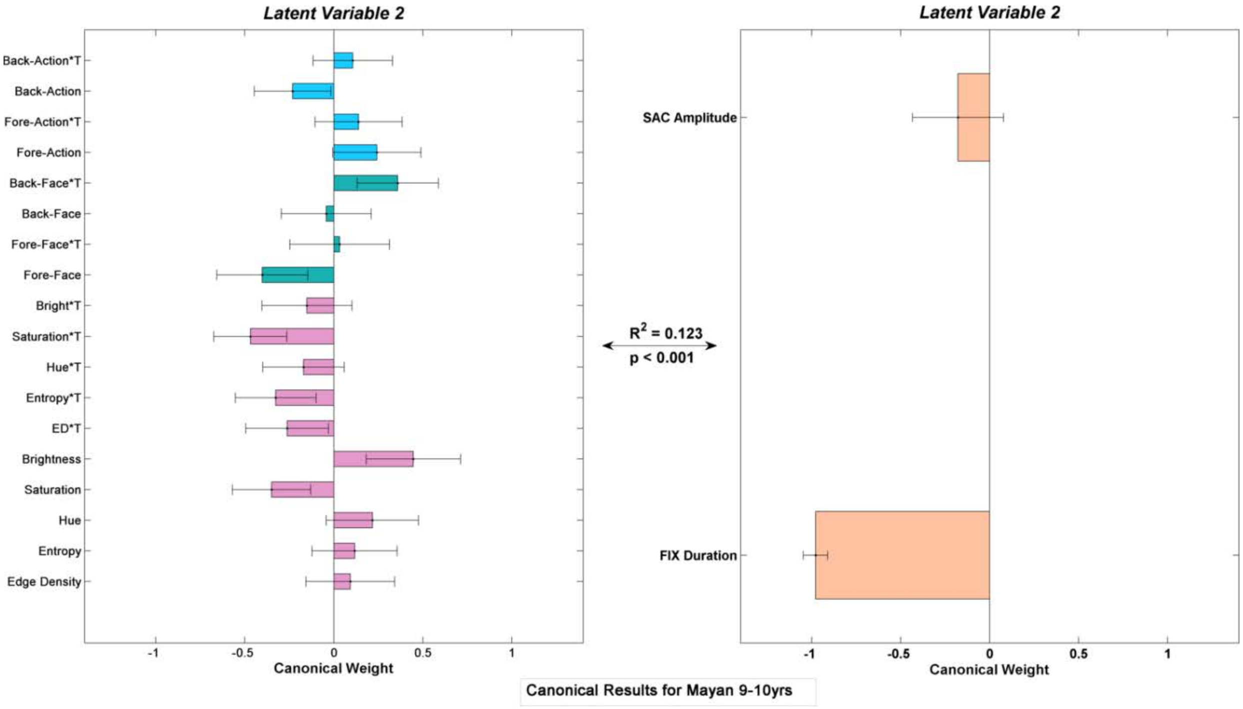

Figure S12. Second component of the canonical results for Mayan older children.

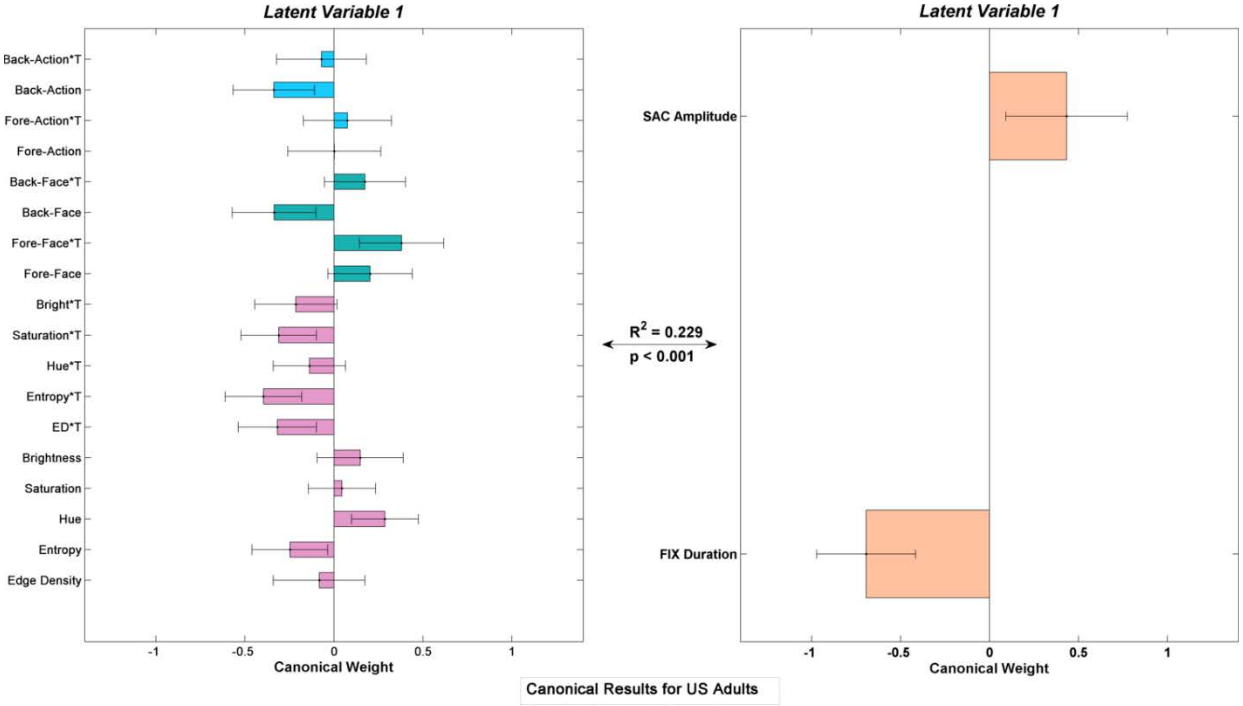

Figure S13. First component of the canonical results for the US adults.

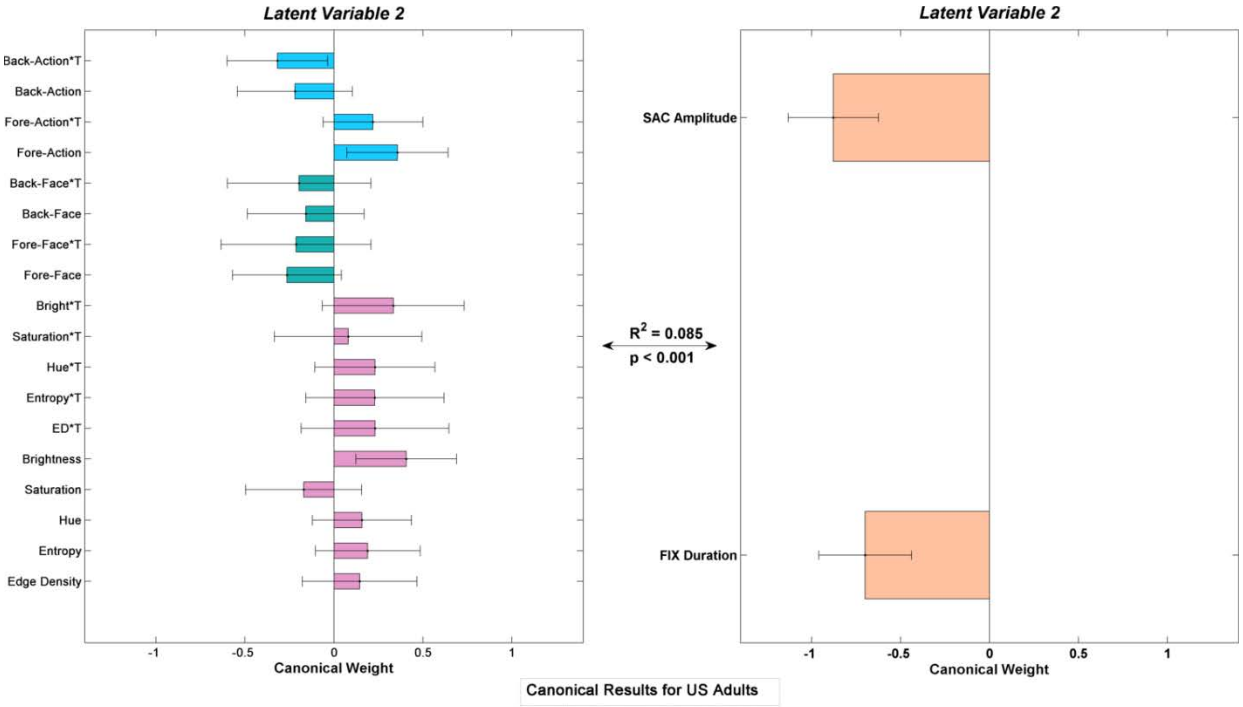

Figure S14. Second component of the canonical results for the US adults.

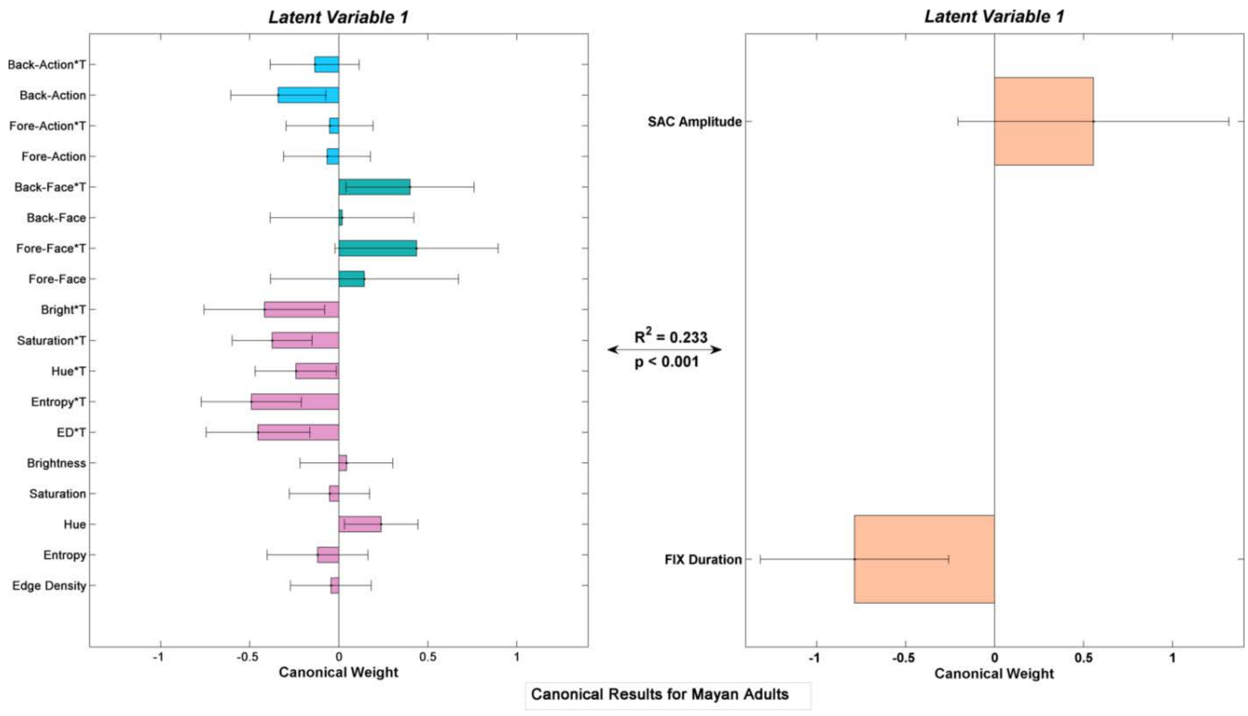

Figure S15. First component of the canonical results for Mayan adults.

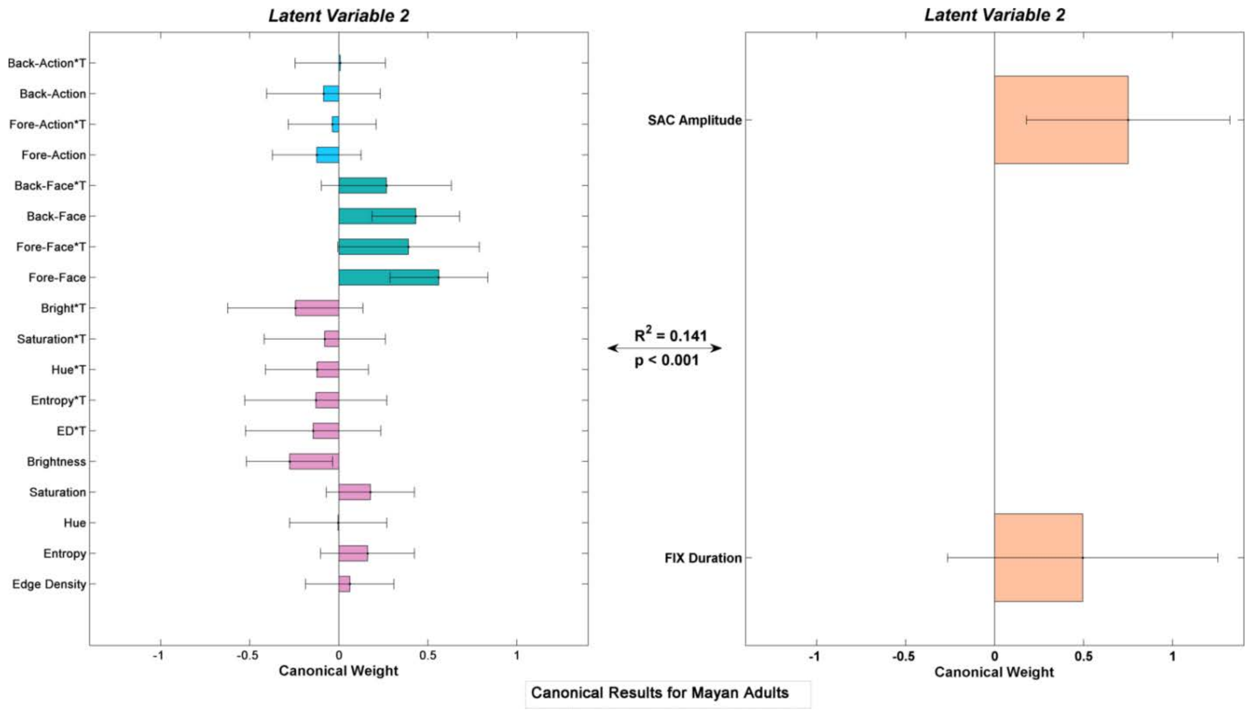

Figure S16. Second component of the canonical results for Mayan adults.

Reproducibility of the canonical correlation models

One caveat of the results from the multivariate analysis is that the sample in each age-culture group is small (11 or 12 participants in each cell). Small samples have the potential problems of low power and/or sensitivity to outliers, which could result in lack of replicability. Since we are finding reliable differences in eye-movement patterns (Figure 5), by definition we do not have a power issue for the effect sizes observed in our data. However, when running a canonical correlation analysis on every age-culture group, the structure of the covariance matrix of the data in that group could be highly influenced by only few participants because of the relatively small number of participants in every age-culture group and thus limit the replicability of our findings. In order to check the stability of the latent variables that emerged from our previous analyses, we randomly split the data in every group into two subgroups of 6 participants (or one subgroup of 6 and one subgroup of 5 in case of the Mayan infants and US adults), and ran the canonical correlation analysis on each subgroup of the participants separately. Then the correlation between the first latent variable of left side of the canonical model (the video-driven features side) from subgroup 1 and the transformed data from subgroup 2 is calculated, where the transformation applied to subgroup 2 is the vector of the canonical weights from subgroup 1. The same correlation is calculated for the first latent variable of the right side of the canonical model (the eye-movement-driven features side). If the two correlations do not have the same sign, an inconsistency in the canonical model for the current data-splitting is recorded, since the pair of latent variables emerged from the canonical correlations from one subgroup does not generalize to the other. The procedure is repeated for all combinations of the data-splitting (12 choose 6, i.e., 924 combinations or 11 choose 6, i.e., 462 combinations, depending on the group) and the proportion of the inconsistent canonical models are calculated. The same exact cross-validation procedure was also done for the second pair of latent variables from the canonical analyses. Table S1 shows what proportion of the data-splitting combinations that yielded the same pair of latent variables for each age-culture group and each latent variable pair. Values closer to 1 suggest more stable covariance matrices (less sensitive to specific participants) for the data in the sampled group. The results show high stability for the first components (latent variable 1)

in all groups, and high to moderate stability (all within an acceptable range) for the second components (latent variable 2). As such, we are not concerned about the non-reproducibility of the latent variables reported in our analysis.

Table S1. Cross-validation results for stability of canonical correlation models.

Age Group	Culture group	Latent Variable 1	Latent Variable 2
Infants	US	0.986	0.732
	Mayan	0.983	0.918
6-7 years-old	US	0.990	0.855
	Mayan	0.977	0.926
9-10 years-old	US	1.000	0.887
	Mayan	1.000	1.000
adults	US	1.000	0.717
	Mayan	0.889	0.919

Computer simulation on scanning scenes using US vs. Mayan eye-movements

The mechanism through which the cultural bifurcation in the eye-movement patterns between US participants and Mayans emerges by the age of 6 is unknown to us. However, we have some speculations as to the possible 'usefulness' of the observed eye-movement pattern by US participants after age 6. We performed a simple computer simulation where the probability of hitting a random event at random times through a 30 second 'view' of a 1440*1080 pixel space when utilizing US, Mayan, or random eye-movement patterns was simulated. The results (Table S2) showed that while using both the US and Mayan eye-movements was more efficient than chance to efficiently cover the 'field of view', the US pattern of dependent saccades and fixations was slightly but significantly more efficient than the Mayan's for the computer to scan the field of view.

Table S2. Hit rates from the computer simulation of hitting a random event in the visual field while using US, Mayan, or random fixations and saccades. In the two top rows all of the fixations were used. In the two bottom rows only fixations from the active foreground and active background were used. A hit is counted when the event (a random pixel) happens to fall into within 2 degrees of a fixation. Every fixation reduces the remaining time of view equal to the fixation duration. The process keeps going until the 30 seconds is finished, and the percentage of hits is recorded. This process was simulated 10,000 times with resampling (with replacement) from the fixation durations and saccade amplitudes from each of the groups.

**Fixations used**	**Chance of Hit**
US (All)	**6.2%***
Mayan (All)	5.9%*
Random	5.3%
US (Social)	**6.2%***
Mayan (Social)	5.9%*

Note: * shows $p<0.05$ from Wilcoxon signed-rank test when compared to Random. Bold font shows $p<0.05$ from Wilcoxon signed-rank test when compared to Mayan.
